# Mind-controlled transgene expression by a wireless-powered optogenetic designer cell implant

**DOI:** 10.1038/ncomms6392

**Published:** 2014-11-11

**Authors:** Marc Folcher, Sabine Oesterle, Katharina Zwicky, Thushara Thekkottil, Julie Heymoz, Muriel Hohmann, Matthias Christen, Marie Daoud El-Baba, Peter Buchmann, Martin Fussenegger

**Affiliations:** 1 Department of Biosystems Science and Engineering, ETH Zurich, Mattenstrasse 26, CH-4058 Basel, Switzerland; 2 Département Génie Biologique, Institut Universitaire de Technologie (IUTA), 74 Boulevard Niels Bohr, F-69622 Villeurbanne, France; 3 Faculty of Science, University of Basel, Mattenstrasse 26, CH-4058 Basel, Switzerland

## Abstract

Synthetic devices for traceless remote control of gene expression may provide new treatment opportunities in future gene- and cell-based therapies. Here we report the design of a synthetic mind-controlled gene switch that enables human brain activities and mental states to wirelessly programme the transgene expression in human cells. An electroencephalography (EEG)-based brain–computer interface (BCI) processing mental state-specific brain waves programs an inductively linked wireless-powered optogenetic implant containing designer cells engineered for near-infrared (NIR) light-adjustable expression of the human glycoprotein SEAP (secreted alkaline phosphatase). The synthetic optogenetic signalling pathway interfacing the BCI with target gene expression consists of an engineered NIR light-activated bacterial diguanylate cyclase (DGCL) producing the orthogonal second messenger cyclic diguanosine monophosphate (c-di-GMP), which triggers the stimulator of interferon genes (STING)-dependent induction of synthetic interferon-β promoters. Humans generating different mental states (biofeedback control, concentration, meditation) can differentially control SEAP production of the designer cells in culture and of subcutaneous wireless-powered optogenetic implants in mice.

## Abstract (web summary)

Brain–machine interfaces offer the possibility of controlling prosthetic devices using changes in brain activity. Folcher *et al.* couple such a system wirelessly to an optogenetic implant in mice to control expression of a transgene, demonstrating its potential for mind-controlled drug delivery.

## Graphical abstract

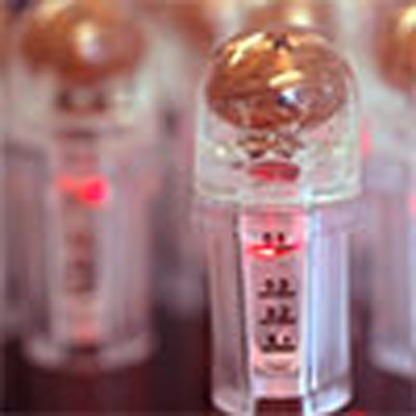

Mammalian synthetic biology has significantly advanced the design of gene switches that are responsive to traceless cues such as light^1^,^2^, gas^3^ and radio waves^4^, complex gene circuits, including oscillators^5^,^6^, cancer-killing gene classifiers^7^,^8^ and programmable biocomputers^9^, as well as prosthetic gene networks^10^ that provide treatment strategies for gouty arthritis^11^, diabetes^1^,^12^ and obesity^13^. Akin to synthetic biology promoting prosthetic gene networks for the treatment of metabolic disorders^1^,^11^,^12^,^13^, cybernetics advances the design of functional man–machine interfaces in which brain–computer interfaces (BCI)^14^,^15^ process brain waves to control electromechanical prostheses, such as bionic extremities^16^ and even wheel chairs^17^. The advent of synthetic optogenetic devices that use power-controlled, light-adjustable therapeutic interventions^18^ will enable the merging of synthetic biology with cybernetics to allow brain waves to remotely control the transgene expression and cellular behaviour in a wireless manner.

Optogenetic devices operating in the near-infrared (NIR) spectral range combine high tissue penetration power with negligible phototoxicity^19^,^20^. The phototrophic bacterium *Rhodobacter sphaeroides* is able to capture NIR light with the multidomain protein BphG1, which contains an amino-terminal (N-terminal) NIR light sensor and carboxyl-terminal diguanylate cyclase (DGC) domain, as well as phosphodiesterase (PDE) activities, to control the level of the ubiquitous bacterial second messenger cyclic diguanosine monophosphate (c-di-GMP)^21^ and orchestrate the environmental light-triggered transition from motile cells to biofilm-forming communities^22^. Stimulator of interferon genes (STING)^23^ was recently identified as a novel player in the human innate immunity that functions as a cyclic di-nucleotide sensor (cGAMP, c-di-AMP, c-di-GMP) to detect the presence of cytosolic DNA via cyclic-GMP–AMP (cGAMP) synthase (cGAS)-mediated production of cGAMP^24^, as well as second messengers (c-di-AMP, c-di-GMP) released from intracellular pathogens^25^,^26^,^27^. Activated STING specifies the phosphorylation of the interferon-regulatory factor 3 (IRF3) (ref. 28) by tank-binding kinase 1, which results in the nuclear translocation of IRF3, binding to IRF3-specific operators and induction of type I interferon promoters^25^,^28^. In this study, we rewire BCI-triggered NIR light-based induction of c-di-GMP production by BphG1 variants to c-di-GMP-dependent STING-driven activation of optimized interferon-responsive promoters to enable mind-controlled transgene expression in mammalian designer cells inside subcutaneous wireless-powered optogenetic implants in mice. Cybernetic control of synthetic gene networks in designer mammalian cells may pave the way for mind-genetic interfaces in future treatment strategies.

## Results

### c-di-GMP, an orthogonal second messenger in mammalian cells

**Figure 1:**
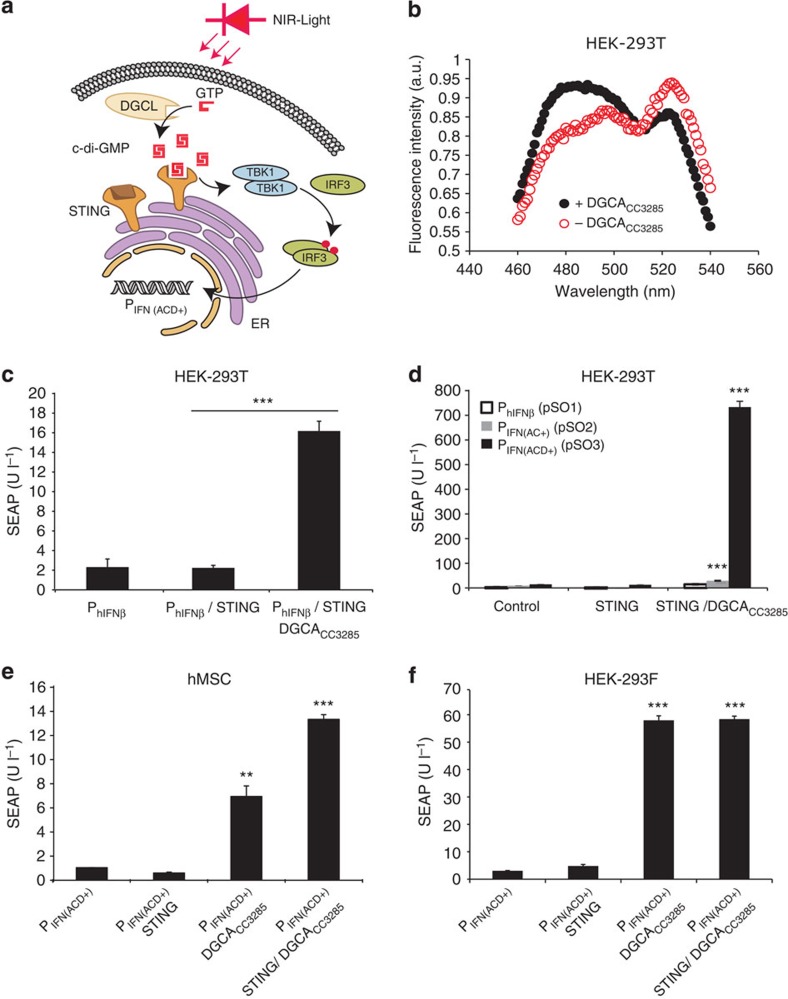
Synthetic mammalian c-di-GMP-based second messenger pathway. (**a**) Schematic representation of the synthetic mammalian optogenetic signalling pathway. NIR light activates an engineered light-dependent bacterial phytochrome-associated DGCL, which converts GTP to the orthogonal second messenger cyclic diguanylate monophosphate (c-di-GMP). c-di-GMP binds and activates STING at the endoplasmic reticulum (ER) and specifies tank-binding kinase 1 (TBK1)-mediated phosphorylation of IRF3 (red dots). Phosphorylated IRF3 translocates to the nucleus, binds IRF3-specific operators and induces the optimized type-1 interferon promoters (P_IFN(ACD+)_). (**b**) FRET-based detection of c-di-GMP in HEK-293T cells containing the FRET biosensor plasmid pKZY81 (P_SV40_-mYPet-YcgR-mCYPet-pA) (co)-transfected with or without the DGCA_CC3285_-expression vector pZKY121 (P_SV40_-DGCA_CC3285_-pA). After excitation at 425 nm, FRET emission was scanned from 460 to 560 nm at 2-nm intervals. (**c**) c-di-GMP-based activation of STING-mediated induction of the hIFN-β promoter (P_hIFNß_). A total of 5 × 10^5^ HEK-293T cells were (co)-transfected with different combinations of the constitutive *Caulobacter crescentus* DGCA (DGCA_CC3285_) expression vector pZKY121 (P_SV40_-DGCA_CC3285_-pA), the constitutive STING expression vector pSTING (P_hCMV_-STING-pA) and pSO1 (P_hIFNß_-SEAP-pA) to encode the human placental secreted alkaline phosphatase (SEAP) driven by the hIFN-β promoter (P_hIFNß_). SEAP expression was profiled in the culture supernatant after 48 h. HEK-293T cells require ectopic expression of STING to complement the corresponding endogenous mammalian pathway. (**d**) Comparative performance analysis of P_hIFNß_ variants (P_hIFNß_, P_IFN(AC+),_ P_IFN(ACD+)_). A total of 1 × 10^6^ HEK-293T cells were co-transfected with the DGCA_CC3285_-expression vector pKZY121, pSTING and pSO1 (P_hIFNß_-SEAP-pA), pSO2 (P_IFN(AC+)_-SEAP-pA) or pSO3 (P_IFN(ACD+)_-SEAP-pA), and SEAP levels were quantified in the culture supernatant after 48 h. Control populations were transfected without pKZY121 or without pKZY121 and pSTING. (**e**,**f**) Validation of the synthetic mammalian c-di-GMP-based second messenger pathway in human stem cells (hMSCs; **e**) and HEK-293T-derived serum-free suspension cultures (HEK-293F; **f**). A total of 5 × 10^4^ hMSCs or HEK-293F cells were (co)-transfected with combinations of the DGCA_CC3285_-expression vector pZKY121 (P_SV40_-DGCA_CC3285_-pA), the STING expression vector pSTING (P_hCMV_-STING-pA) and pSO3 (P_IFN(ACD+)_-SEAP-pA). SEAP production was assessed in the culture supernatant after 48 h. Data are mean±s.d.; statistics by two-tailed *t*-test; *n*=6, triplicate experiments, **P<0.01, ***P<0.001.

The design of the synthetic mammalian optogenetic signalling pathway included the combination of the NIR light-activated DGCL (pSO4, P_hCMV_-DGCL-pA), a PDE-deficient *Rhodobacter sphaeroides* BphG1 variant that produces the orthogonal second messenger c-di-GMP, and STING (pSTING, P_hCMV_-STING-pA) to sense intracellular c-di-GMP levels and manage dose-dependent activation of an engineered interferon-β promoter P_IFN(ACD+)_, thereby driving the transcription of a specific transgene (pSO3, P_IFN(AC+)_-SEAP-pA; Fig. 1a). To confirm that the prokaryotic DGCLs can produce the bacterial second messenger c-di-GMP in living mammalian cells, we co-transfected HEK-293T cells with DGCA_CC3285,_ which is feedback inhibited by c-di-GMP (pZKY121, P_SV40_-DGCA_CC3285_-pA) and an intracellular fluorescence resonance energy transfer (FRET)-based c-di-GMP biosensor (pKZ81, P_SV40_-mYPet-YcgR-mCyPet; Fig. 1b). Constitutive expression of the DGCL and the resulting c-di-GMP pool had no negative impact on the viability (Supplementary Fig. 1a) or metabolic capacity (Supplementary Fig. 1b) of the engineered mammalian cells.

### Rewiring c-di-GMP to the STING-specific signalling cascade

To demonstrate that c-di-GMP can be functionally rewired for STING-mediated activation of P_hIFNß_-driven transgene expression, we co-transfected HEK-293T cells with pZKY121 (P_SV40_-DGCA_CC3285_-pA), pSTING (P_hCMV_-STING-pA) and pSO1 (P_hIFNß_-SEAP-pA) and scored SEAP production after 48 h (Fig. 1c). When the two different human codon-optimized c-di-GMP-specific phophodiesterases PDE_yahA_ (pKZY119, P_SV40_-PDE_yahA_-pA) and PDE_TBD1265_ (pKZY120, P_SV40_-PDE_TBD1265_-pA) were co-transfected into HEK-293T cells co-transfected with pZKY121, pSTING and pSO1, the PDEs reduced the intracellular c-di-GMP inducer pool, resulting in decreased P_hIFNß_-driven SEAP expression (Supplementary Fig. 2). The use of P_hIFNß_ variants with optimized IRF3 operator sites (pSO1, P_hIFNß_-SEAP-pA; pSO2 P_IFN(AC+)_-SEAP-pA; pSO3, P_IFN(ACD+)_-SEAP-pA) (Supplementary Fig. 3) resulted in an up to 60-fold increase in the response to DGCA_CC3285_-produced c-di-GMP (Fig. 1d).

Control experiments profiling human interferon-β (hIFN-β) in the culture supernatant of pZKY121/pSTING/pSO3-co-transfected HEK-293T cells showed no detectable hIFN-β levels in the presence of an activated c-di-GMP-based second messenger signalling pathway (Supplementary Fig. 4a). In addition, paracrine hIFN-β had no effect on STING-mediated activation of P_IFN(ACD+)_ (Supplementary Fig. 4b). Interestingly, when testing the synthetic second messenger pathway containing the optimal P_IFN(ACD+)_ promoter (pZKY121, P_SV40_-DGCA_CC3285_-pA; pSTING, P_hCMV_-STING-pA; pSO3, P_IFN(ACD+)_-SEAP-pA) in human stem cells (hMSCs) or HEK-293F cells, c-di-GMP-induced STING-mediated P_IFN(ACD+)_ activation was cell line dependent (Fig. 1e,f). While the ectopic expression of STING significantly increased SEAP expression in hMSCs (Fig. 1e), heterologous STING expression in HEK-293F cells was dispensable, consequently significantly simplifying the synthetic optogenetic device to a two-component configuration (DGCL and P_IFN(ACD+)_; Fig. 1f). HEK-293F is a GMP-compliant derivative of the Food and Drug Administration-licensed HEK-293 cell line that grows in serum-free suspension cultures, an important asset for the biopharmaceutical manufacturing^29^ and maintenance of cells inside implantable microcontainers.

### A NIR light-sensitive transcription control device

**Figure 2:**
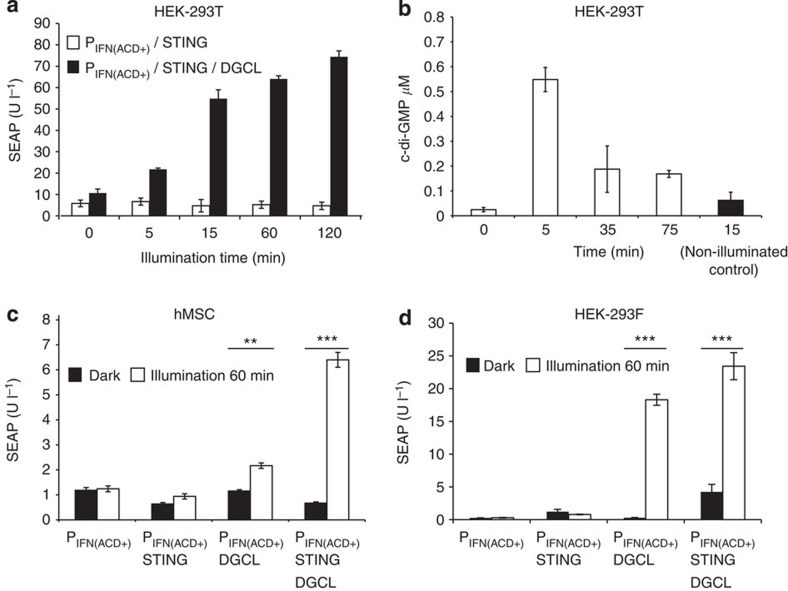
Design and characterization of the synthetic mammalian optogenetic pathway. (**a**) A total of 5 × 10^4^ HEK-293T cells were co-transfected with pSO4 (P_hCMV_-DGCL-pA), pSTING (P_hCMV_-STING-pA) and pSO3 (P_IFN(ACD+)_-SEAP-pA) and illuminated with NIR light (700 nm) for different periods of time before profiling SEAP in the culture supernatant after 24 h. (**b**) Quantification of NIR light-induced c-di-GMP in HEK-293T cells. A total of 5 × 10^4^ HEK-293T cells were transfected with pSO4 and illuminated for 15 min with NIR light (700 nm). Intracellular c-di-GMP levels were then profiled for different periods of time. Non-illuminated cell populations were used as a negative control. (**c**,**d**) Validation of the synthetic mammalian optogenetic pathway in human stem cells (hMSCs; **c**) and HEK-293T-derived serum-free suspension cultures (HEK-293F; **d**). A total of 5 × 10^4^ hMSCs (**c**) or HEK-293F cells (**d**) were (co)-transfected with combinations of the NIR light-activated DGCL expression vector pSO4 (P_hCMV_-DGCL-pA), the STING expression vector pSTING (P_hCMV_-STING-pA) and pSO3 (P_IFN(ACD+)_-SEAP-pA) to encode the human placental SEAP driven by the engineered hIFN-β promoter (P_IFN(ACD+)_) and illuminated for 1 h with NIR light (700 nm). SEAP production was then assessed in the culture supernatant after 24 h. Data are mean±s.d.; statistics by two-tailed *t*-test; *n*=6, triplicate experiments, **P<0.01, ***P<0.001.

**Figure 3:**
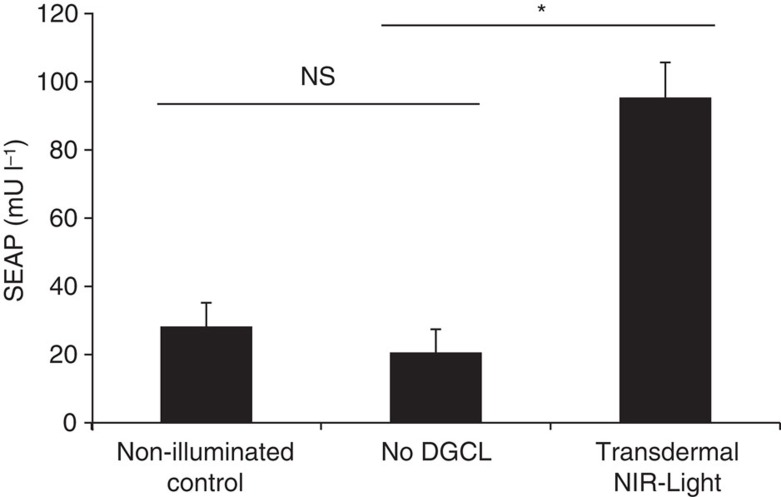
Percutaneous control of NIR light-inducible transgene expression in mice. Hollow-fibre implants containing 5 × 10^4^ HEK-293T cells transgenic for pSO4 (P_hCMV_-DGCL-pA), pSTING (P_hCMV_-STING-pA) and pSO3 (P_IFN(ACD+)_-SEAP-pA) were subcutaneously inserted into wild-type mice, which were then percutaneously illuminated for 2 h with NIR light (700 nm). SEAP levels were profiled in the bloodstream of the treated animals after 24 h. Non-illuminated mice or animals implanted with DGCL-deficient designer cells were used as negative controls. Data are mean±s.d.; statistics by two-tailed *t*-test; *n*=5 mice. *P<0.05; NS, not significant.

To render the synthetic mammalian c-di-GMP pathway responsive to NIR light, we co-transfected HEK-293T cells with DGCL (pSO4, P_hCMV_-DGCL-pA), a truncated PDE domain-deficient *R. sphaeroides* BphG1 variant, pSO3 (P_IFN(ACD+)_-SEAP-pA) and pSTING (P_hCMV_-STING-pA) (Fig. 1a). We then illuminated the engineered cells for different periods of time with an NIR LED panel and recorded the corresponding SEAP expression profiles (Fig. 2a). NIR-light-controlled transgene expression was adjustable (Fig. 2a) and displayed rapid reversible c-di-GMP ON/OFF-response profiles in the range of minutes that were characterized by fast synthesis kinetics (Fig. 2b) and a rapid decrease of intracellular c-di-GMP levels promptly after NIR-light switch-off, possibly due to the presence of numerous endogenous PDEs (Fig. 2b). The synthetic optogenetic signalling pathway was compatible with different mammalian cell lines, including hMSCs (Fig. 2c), as well as HEK-293F (Fig. 2d). To validate the NIR light remote-controlled transgene expression *in vivo*, we implanted hollow-fibre microcontainers enclosing pSO3-/pSO4-/pSTING-transgenic HEK-293T cells subcutaneously into mice, transdermally illuminated the treated animals with NIR light and profiled the resulting SEAP levels in their bloodstream (Fig. 3).

### Mind-controlled transgene expression in mammalian cells

**Figure 4:**
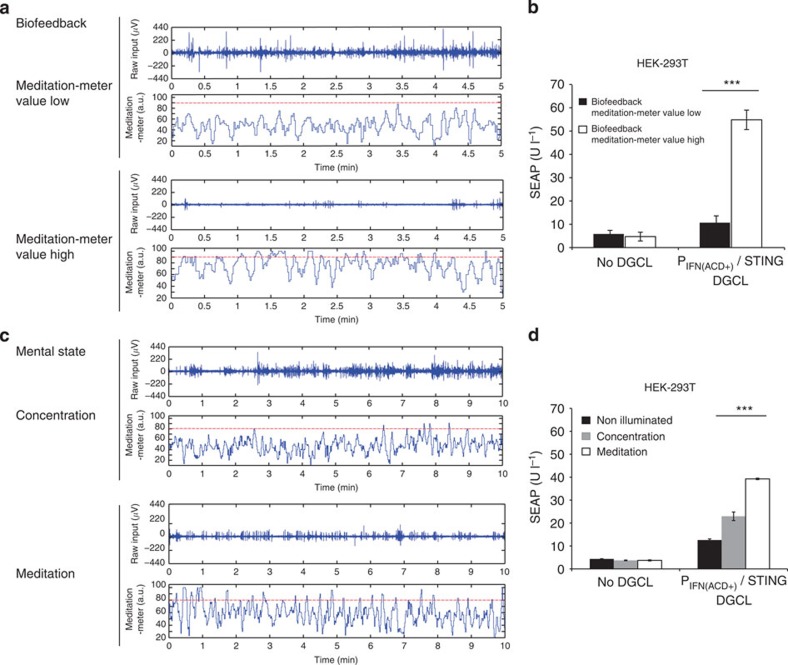
The mind-controlled electro-optogenetic interface. (**a**,**b**) Biofeedback-controlled transgene expression switch in HEK-293T cells transgenic for DGCL (pSO4, P_hCMV_-DGCL-pA), STING (pSTING) and P_IFN(ACD+)_-driven SEAP expression (pSO3, P_IFN(ACD+)_-SEAP-pA). (**a**) A human subject wearing an EEG headset, which captured brain-wave activities (raw input (μV)) and identified mental state-specific electrical patterns as discrete meditation-meter values (0–100), intentionally trained his/her mindset to maintain the biofeedback-derived meditation-meter value below (meditation-meter value low) or above (meditation-meter value high) a threshold value of 90 (dotted red line) by following the meditation-meter value displayed on the LCD computer screen in a biofeedback-controlled manner in real time. (**b**) Mind-controlled biofeedback-derived meditation-meter values above 90 triggered NIR light illumination of the engineered HEK-293T cells and programmed the optogenetic device of these designer cells to express SEAP. Meditation-meter values below 90 did not illuminate the designer cells, resulting in basal SEAP expression comparable to that of isogenic control HEK-293T populations deficient in DGCL expression. (**c**,**d**) Mental states, such as concentration and meditation, controlled the transgene expression in HEK-293T cells transgenic for DGCL (pSO4, P_hCMV_-DGCL-pA), STING (pSTING) and P_IFN(ACD+)_-driven SEAP expression (pSO3, P_IFN(ACD+)_-SEAP-pA). (**c**) A human subject wearing an EEG headset, which captured brain-wave activities (raw input (μV)) and identified mental state-specific electrical patterns as discrete meditation-meter values (0–100), generated mental states, such as concentration (computer gaming) and meditation (relaxation), without visual inspection of the displayed meditation-meter values (no biofeedback). The subject’s mental state maintained the meditation-meter value below (concentration) or above (meditation) a threshold value of 80 (dotted red line). (**d**) Whenever the mental state drove the meditation-meter value above a threshold of 80, the BCI triggered an NIR light pulse that illuminated the engineered HEK-293T cells. The designer cells integrated the NIR light pulses and produced a sustained high (meditation) or low (concentration) SEAP expression response. Isogenic non-illuminated HEK-293T populations and designer cells deficient in DGCL expression were used as negative controls. Data are mean±s.d.; statistics by two-tailed *t*-test; *n*=6. ***P<0.001.

**Figure 5:**
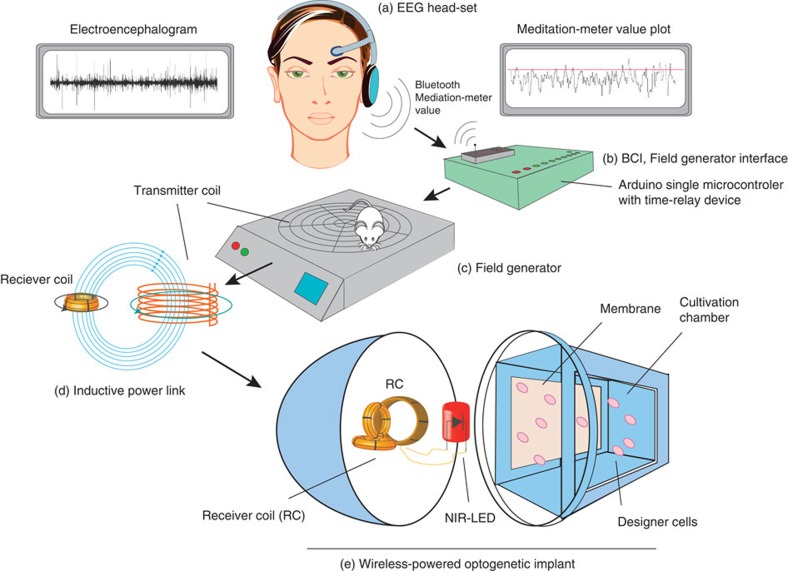
Schematic representation of mind-controlled transgene expression. The mind-controlled transgene expression device consisted of (**a**) an EEG headset that captured brain-wave activities (the encephalogram), identified mental state-specific electrical patterns (biofeedback, concentration, meditation) and processed discrete meditation-meter values (0–100; meditation-meter value plot), which were transmitted via Bluetooth to (**b**) the Arduino single-board microcontroller with a time-relay device and switching the (**c**) field generator ON and OFF. This BCI (**a**–**c**) controlled (**d**) the TC (**c**,**d**) of the field generator, which inductively coupled with the (**d**,**e**) receiver coil (RC) of the (**e**) wireless-powered optogenetic implant. (**e**) The NIR light LED illuminated the culture chamber of the wireless-powered optogenetic implant and programmed the designer cells to produce SEAP, which diffused through the semi-permeable membrane. The blood SEAP levels of mice with subcutaneous wireless-powered optogenetic implants containing designer cells that were freely moving on the field generator could be modulated by the human subject’s mindset in a wireless, remote-controlled manner. (See Supplementary Fig. 5 for a schematic of the electronic components).

By connecting the mind-triggered electrophysiological signals via an electroencephalography (EEG)-based BCI to the synthetic mammalian NIR light-triggered optogenetic signalling pathway, we designed a mind-genetic interface that uses brain waves to remotely control target gene transcription wirelessly. Therefore, an EEG headset was used to capture brain-wave activities and identify mental state-specific electrical patterns (discrete meditation-meter values, 0–100) resulting from self-trained biofeedback (maintaining the observed meditation-meter value within a desired range^30^), concentration (computer gaming) or meditation (relaxation; Fig. 4; Fig. 5a,b; Supplementary Fig. 5a,b). This BCI was set to power the NIR light and control the illumination time in response to meditation-meter threshold (Fig. 4). Human subjects wearing the EEG headset were thus able to intentionally programme the transgene expression of cultured pSO3-/pSO4-/pSTING-transgenic HEK-293T cells by mental states, such as biofeedback (Fig. 4a,b), concentration (Fig. 4c,d) or meditation (Fig. 4c,d). Non-illuminated pSO3-/pSO4-/pSTING-transgenic HEK-293T cells and DGCL-deficient pSO3-/pSTING-transgenic HEK-293T cells were used as negative controls (Fig. 4b,d).

### A mind-controlled wireless-powered implant in mice

**Figure 6:**
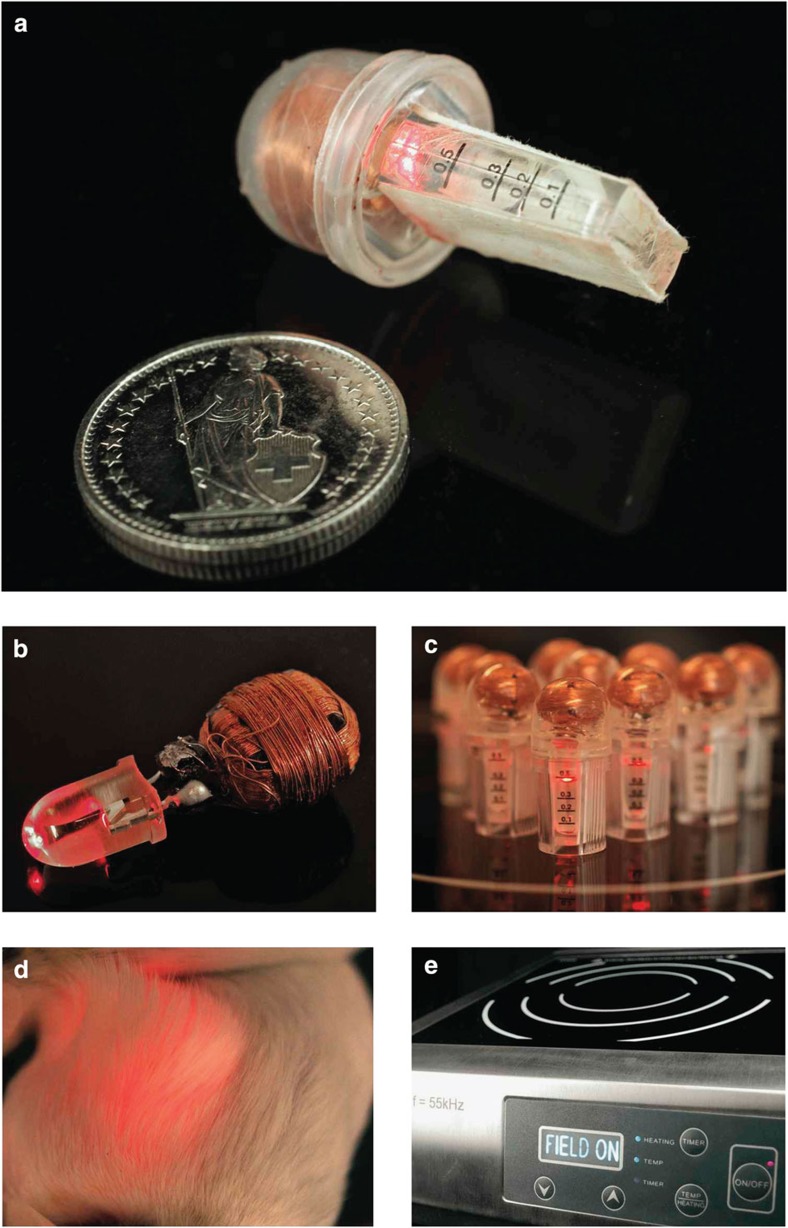
Wireless-powered optogenetic implant. (**a**) Wireless-powered implant on the field generator with an illuminated NIR LED. A 1 CHF coin (23 mm in diameter) serves as a size indicator. The 0.5-ml cultivation chamber containing semi-permeable PES membranes on both sides was moulded to a spherical polycarbonate cap contain a PDMS-sealed three-dimensional (3D) receiver antenna wired to the NIR-LED. (**b**) 3D receiver antenna wired via the receiver circuit (receiver coils, resonance capacitors, Schottky diodes; Supplementary Figs 5 and 11) to the NIR LED. (**c**) Quality-control test of the custom-made wireless-powered optogenetic implants illuminated while standing on the powered field generator. (**d**) Mouse with a subcutaneous wireless-powered optogenetic implant, the activity of which can be observed through the skin. (**e**) Field generator.

For mind-controlled transgene expression in mice, the BCI drove a field generator that wirelessly powered an inductively linked NIR light-containing implant enclosing pSO3-/pSO4-transgenic HEK-293F cells in serum-free suspension cultures (Fig. 5; Supplementary Fig. 5). The wireless-powered optogenetic implant (Fig. 5e; Fig. 6a; Supplementary Fig. 5e) consisted of a cultivation chamber with a semi-permeable <300 kDa-cutoff membrane (Fig. 5e; Fig. 6a) to provide the molecular interface between the designer cells and the animal’s peripheral circulation and a sealed electronic compartment (Fig. 5e; Fig. 6a; Supplementary Fig. 5e) in which the NIR LED was connected to the power-receiving antenna containing three orthogonal receiver coils (Fig. 5e; Fig. 6b; Supplementary Fig. 5e), which continuously powered the implant NIR LED (Fig. 5e; Fig. 6a–c; Supplementary Fig. 5e) as the animals moved freely (Fig. 6d) on the field generator (Fig. 5c; Fig. 6e; Supplementary Fig. 5c). The wireless-powered optogenetic implant electronics were validated by scoring the coupling intensity above the field generator (Supplementary Fig. 6) and confirming the molecular cutoff for viruses and bacteria (Supplementary Fig. 7).

**Figure 7:**
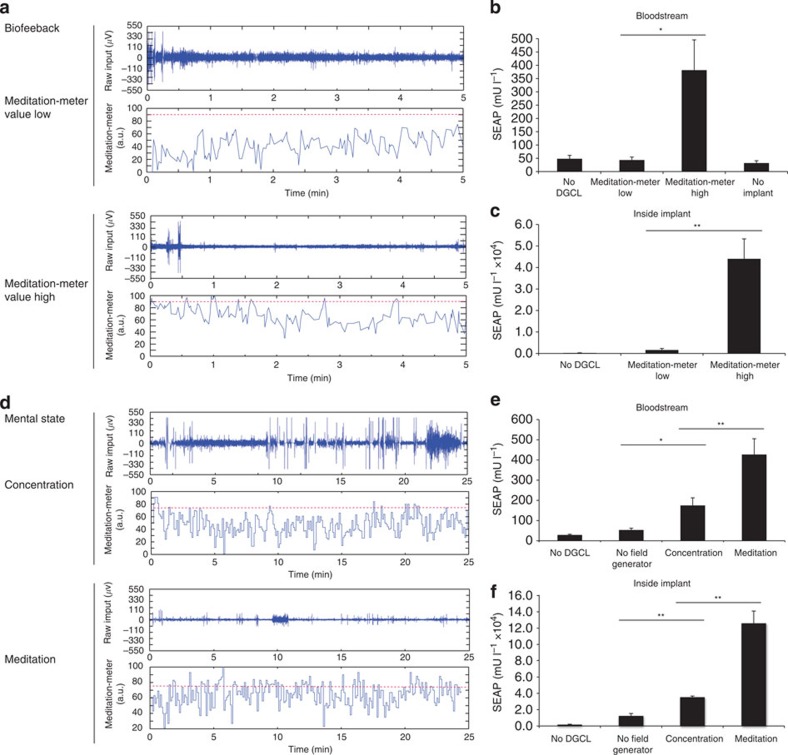
Mind-controlled wireless-powered optogenetic implant in mice. (**a**–**c**) Biofeedback-controlled transgene expression in HEK-293F cells transgenic for DGCL (pSO4) and P_IFN(ACD+)_-driven SEAP expression (pSO3) contained in a wireless-powered optogenetic implant (Fig. 5, Supplementary Fig. 5). (**a**) A human subject wearing an EEG headset capturing brain-wave activities (raw input (μV)) and providing discrete meditation-meter values (0–100), trained his/her mindset to maintain the biofeedback-derived meditation-meter value below (meditation-meter value low) or above (meditation-meter value high) a threshold value of 90 (dotted red line) (**b**,**c**) Mind-controlled meditation-meter values above 90 activated a field generator, inductively powered the subcutaneous wireless optogenetic implant inside the mice freely moving in the field generator, illuminated the culture chamber, thereby programming the designer cells to secrete SEAP that was measured in the animals’ bloodstream (**b**) and the implant chamber. (**c**) Isogenic DCL-deficient HEK-293F cells and mice without implants served as negative controls. (**d**–**f**) Mental states controlling transgene expression in HEK-293F cells transgenic for DGCL (pSO4) and P_IFN(ACD+)_-driven SEAP expression (pSO3) contained in a wireless-powered optogenetic implant. (**d**) A human subject wearing an EEG headset, capturing brain-wave activities (raw input (μV)) and providing discrete meditation-meter values (0–100), generated specific mental states, such as concentration (computer gaming) and meditation (relaxation), without visual inspection of the displayed meditation-meter values (no biofeedback). The subject’s mental state maintained the meditation-meter value below (concentration) or above (meditation) a threshold value of 75 (dotted red line). (**e**,**f**) Mind-controlled meditation-meter values above 75, activated a field generator, inductively powered the subcutaneous wireless optogenetic implant inside the mice freely moving in the field generator, illuminated the culture chamber, thereby programming the designer cells to secrete SEAP that was measured in the animals’ bloodstream (**e**) and the implant chamber (**f**). Isogenic DCL-deficient HEK-293F cells and treated mice not exposed to the field generator were used as negative controls. Data are mean±s.d.; statistics by two-tailed *t*-test; *n*=5 mice. *P<0.05, **P<0.01.

When the pSO3-/pSO4-transgenic HEK-293F-containing wireless-powered optogenetic implants were placed subcutaneously on the back of wild-type mice moving on the field generator, the animals’ blood SEAP levels could be controlled by the human subject’s mental states, such as biofeedback (Fig. 7a–c), concentration (Fig. 7d–f) or meditation (Fig. 7d–f). Mind-triggered NIR light activation could be observed through the mouse skin in real time (Fig. 6d). Illuminated DGCL-deficient pSO3-transgenic HEK-293F implants (Fig. 7b,c,e,f) and non-illuminated pSO3-/pSO4-transgenic HEK-293F implants (Fig. 7e,f) were used as negative controls. Following removal of the wireless-powered optogenetic implants, the blood SEAP levels of the animals dropped rapidly (Supplementary Fig. 8) and high-level SEAP production of the designer cells inside the implant was confirmed (Fig. 7c,f).

## Discussion

Cybernetics has pioneered mind-controlled electromechanical man–machine interfaces that allow brain activities to intentionally control bionic prostheses^16^, and optogenetics has established electromolecular machine–man interfaces that enable light-controlled therapeutic interventions by modulating brain^31^, heart^32^ and gene activities^1^. By combining cybernetics with optogenetics, we now provide the missing link enabling mental states such as biofeedback, concentration and meditation to directly control the transgene expression in living cells and mammals. An ideal optogenetic device to interface with the BCI with transgene expression would have a simple design, be insensitive to pleiotropic input and provide robust, adjustable, reversible and rapid ON/OFF-switching profiles in response to light with deep-tissue penetration, negligible phototoxicity and address a chromophore that is available in the peripheral circulation. The NIR light-triggered synthetic optogenetic signalling pathway developed herein meets these criteria at a high standard. In particular, c-di-GMP is a mammalian cell-compatible orthogonal second messenger that is produced from intracellular GTP within minutes after NIR light illumination by ectopically expressed DGCL using biliverdin as the chromophore, a haem catabolic product that is abundant in mammalian circulation.

In addition, the endogenous c-di-GMP sensor STING rewires illumination to transcription by managing the activation of the engineered target promoters. Because the availability and abundance of the STING signalling componentry varies between different cell types, the synthetic optogenetic signalling pathway exhibits cell line-specific variations in performance. For example, while ectopic expression of human STING is essential for the NIR light optogenetic device in HEK-293T and boosts performance in human stem cells, it is dispensable in HEK-293F, which relies on endogenous STING for optimal performance. In addition, because HEK-293-derived cell lines do not produce hIFN-β on activation of the c-di-GMP-based second messenger signalling pathway and are deficient in cGAS-mediated type-I interferon production^24^ and STAT2 and IRF9 (ref. 33) expression, they are unable to trigger and fail to respond to paracrine hIFN-β stimulation. Furthermore, the molecular cutoff of the cultivation chamber of the wireless-powered optogenetic device protects the designer cells from intracellular pathogens, making the synthetic optogenetic signalling pathway exclusive for NIR light input.

Currently available optogenetic devices programme the behaviour of implanted designer cells by percutaneous illumination using an extracorporeal light source^1^,^2^,^32^. Although NIR light, which is known for its deep-tissue penetration, was able to programme the product-gene expression of designer cells subcutaneously implanted into mice, we also developed a self-sufficient, removable wireless-powered optogenetic implant to combine placement flexibility and daylight insensitivity with optimal designer cell containment, maximum treatment compliance and host mobility. Wireless-powered optogenetic implants provide a highly modular interface that couples electronics with living cells and enables electronic devices to directly and remotely control gene expression. When coupled to brain activities, such electrogenetic devices provide mind-genetic interfaces that add a new dimension to state-of-the-art electronic-mechanical implants, such as heart and brain pacemakers^34^, cochlear hearing aids^35^,^36^, eye prostheses^37^, insulin-releasing micropumps^36^ and bionic extremities^16^. Here we demonstrated that the transgene expression in mammalian cells and mice can be modulated by three different mental states: biofeedback, concentration and meditation. Far into the future, patients may either learn to generate specific mental states (for example, pain relief^38^), locked-in syndrome^15^,^39^ programming or having disease-related brain activities (for example, epilepsy^40^,^41^, neurodegenerative disorders^42^) close-loop control, therapeutic implants producing corresponding doses of protein pharmaceuticals in real time.

## Methods

### Mind-controlled optogenetic components

Comprehensive design and construction details for all expression vectors are provided in Supplementary Table 1. The integrity of all relevant genetic components was confirmed by sequencing (Microsynth, Balgach, Switzerland). The key plasmids used were as follows: pSO3, which contains SEAP under the control of an optimized hIFN-β promoter (P_IFN(ACD+)_-SEAP-pA; GenBank ID: KM591199); pSO4 that encodes the constitutive expression of a human codon-optimized PDE domain-deficient NIR light-activated DGCL derived from *Rhodobacter sphaeroides* BphG1 (P_hCMV_-DGCL-pA; N-terminal PAS-GAF-PHY-GGDEF portion of BphG1 (Q8VRN4_RHOSH), catalytic DGCL domain GGDEF photoactivated by its cognate PAS-GAF-PHY phytochrome; GenBank ID: (Genbank ID: KM591197)); and pSTING that mediates constitutive expression of mouse STING.

### Cell culture and transfection

Human embryonic kidney cells (HEK-293T, ATCC: CRL-11268) and immortalized hMSCs^43^ were cultivated in Dulbecco’s modified Eagle’s medium (Invitrogen, Basel, Switzerland) supplemented with 10% fetal bovine serum (FBS; cat. no. F7524, lot no. 022M3395, Sigma-Aldrich, Munich, Germany) and 1% (v/v) penicillin/streptomycin solution (Sigma-Aldrich, Munich, Germany). HEK-293-derived FreeStyle 293F suspension cells (HEK-293F; Invitrogen) were grown in FreeStyle 293 expression medium (Invitrogen). All cell types were cultivated at 37 °C in a humidified atmosphere containing 5% CO_2_. Cell concentration and viability were profiled with a CASY Cell Counter and Analyser System Model TT (Roche Diagnostics, Mannheim, Germany). For (co)-transfection, 5 × 10^4^ HEK-293T, HEK-293F or hMSCs were diluted in 0.4 ml of culture medium and seeded per well of a 24-well plate 12 h before (co)-transfection. The cells were then incubated for 6 h with 200 μl of a 1:2 PEI:DNA mixture (w/w) (polyethyleneimine; MW 40,000, Polysciences, Inc., Warrington, USA) containing 1 μg of total DNA (for co-transfections, an equal amount of plasmid DNA was used unless otherwise indicated). After (co)-transfection, the culture medium was replaced, and the engineered cells were used for a dedicated experiment, which was typically analysed for 48 h.

### Production and transduction of lentiviral particles

To produce enhanced yellow fluorescent protein (EYFP)-expressing lentiviral particles, HEK-293T cells (5 × 10^5^ cells per well of a six-well plate) were co-transfected by incubating the cells for 6 h with 200 μl of a 1:2 PEI:DNA mixture containing 1 μg of pLTR-G, which encodes the constitutive expression of the vesicular stomatitis virus G protein^44^, 1 μg of the helper plasmid pCD/NL/BH* (ref. 45) and 2 μg of pNLK8 (5′LTR-ψ^+^-ori_SV40_-cPPT-RRE-P_hEF1α_-EYFP-3′LTR_ΔU3_)^46^. The lentiviral particles were collected from the culture supernatant 48 h after transfection and quantified as described previously^44^,^47^. In brief, lentiviral transduction units were estimated by transduction of 5 × 10^5^ HEK-293T cells with serially diluted lentiviral particles and subsequent quantification of the transduced cells by fluorescence microscopy. To validate the virus-specific 300-kDa molecular weight cutoff of the implant membrane, we sequentially injected 5 × 10^5^ HEK-293F cells and 5 × 10^5^ EYFP-encoding lentiviral particles (24 h later) into the implant and placed the sealed implant in a culture vial containing 5 × 10^5^ HEK-293F cells. After 72 h, EYFP-fluorescent cells were visualized by fluorescence microscopy.

### Fluorescence microscopy

EYFP expression by HEK-293F was visualized using a Leica DM-IL equipped with a DC300 FX camera (Leica Microsystems, Heerbrugg, Switzerland) and a YFP S filter system.

### SEAP assay

Production of human placental SEAP was quantified in culture supernatants according to a *p*-nitrophenylphosphate-based light absorbance time course^48^. SEAP levels of serum, which was isolated from blood samples using microtainer SST tubes (Becton Dickinson, Plymouth, UK), were profiled using a chemiluminescence-based assay (Roche Diagnostics).

### c-di-GMP assay

c-di-GMP was detected in cells using a genetically encoded c-di-GMP-specific FRET biosensor consisting of the central *Salmonella typhimurium*-derived diguanylate receptor domain YcgR flanked by yellow (mYPet) and cyan (mCYPet) fluorescent protein domains (mYPet-YcgR-mCYPet)^49^. The fluorescent protein domains are in closest proximity in the absence of c-di-GMP, with maximal FRET, and the FRET signal dose dependently decreases as c-di-GMP binds YcgR, which alters the relative orientation of the FRET pair mYPet and mCYPet. The FRET biosensor was expressed in pET15b::mYPet-ycgR-mCYPet-transformed *Escherichia coli* and affinity purified via its N-terminal polyhistidine tag^49^.

For the *in vitro* FRET-based analysis of intracellular c-di-GMP levels in mammalian cells, 5 × 10^4^ cells were collected by centrifugation (2 min, 4,500*g*, 20 °C) and lysed in 0.5 ml of ice-cold acetonitrile/CH_3_OH/ddH_2_O (2:2:1, v/v/v) by sequential incubation on ice (15 min) and 95 °C (5 min). The cell lysate was cleared of cell debris by centrifugation (5 min, 14,000*g*, 4 °C) and the supernatant was vacuum dried for 120 min at 40 °C. The pellet was resuspended in 12 μl of PBS (137 mM NaCl, 2.7 mM KCl, 4.3 mM Na_2_HPO_4_, 1.4 mM KH_2_PO_4_; pH 7.4) and then serially diluted in PBS. For FRET-based c-di-GMP quantification, 5 μl of serially diluted mammalian cell extracts and 10 μl of mYPet-YcgR-mCYPet (50 nM, in PBS pH 7.4) were mixed in each well of a 384-well plate, and the FRET ratio of excitation (425 nm) and emission (535 nm) was profiled using an EnVision 2104 multilabel plate reader equipped with a quad monochromator (excitation at 425 nm, emission scan between 460 and 560 nm at 2-nm intervals; PerkinElmer, Waltham, MA, USA). The calibration curve using recombinant myPet-YcgR-mCyPet protein was linear within the range of 10 nM to 1 μM c-di-GMP.

mYPet-YcgR-mCYPet was also used for FRET-based detection of c-di-GMP levels in living mammalian cells. Therefore, HEK-293T cells were (co)-transfected with pKZY81 (P_SV40_-mYPet-YcgR-mCyPet-pA) alone or together with pKZY121 (P_SV40_-DGCA_CC3285_-pA; 4:1 ratio). After 24 h, the transfected cells were washed once with PBS and placed in a black 96-well plate (2.5 × 10^5^ cells per well), and FRET was profiled as described above.

### hIFN-β assay

human IFN-β was quantified by ELISA (VeriKine Human IFN-β ELISA Kit no. 41410; PBL Assay Science, Lausen, Switzerland). Paracrine stimulation of P_IFN(ACD+)_ was tested by transfecting 5 × 10^4^ HEK-293T cells with pSTING and pSO3 (P_IFN(ACD+)_-SEAP-pA), followed by the addition of recombinant hIFN-β (100 units, 1 × 10^4^ units ml^−1^ stock solution in 50 mM NaOAc, 0.1% BSA, pH 5.5; no. 11415-1; PBL, Assay Science). As a positive control for STING activation, 50 μg ml^−1^ DMXAA (5,6-dimethylxanthenone-4-acetic acid, 10 mg ml^−1^ stock solution in dimethylsulphoxide; Santa Cruz Biotechnology, Santa Cruz, CA, USA) was used.

### Optogenetic transgene expression in mammalian cells

HEK-293T/HEK-293F cells transgenic for pSO3, pSO4 and pSTING were cultivated in colourless phenol red-free Dulbecco’s modified Eagle’s medium/FreeStyle 293 expression medium (Invitrogen) supplemented with 25 μM biliverdin hydrochloride (Livchem, Frankfurt am Main, Germany), a haemoglobin catabolite taken up by cells and serving as a DCGL chromophore^50^,^51^,^52^. The 12-well culture plates were placed 7 cm below a custom-designed 3 × 4 LED panel (each LED centred above a single well; *λ*_max_=700 nm, 20 mW sr^−1^; cat. no. ELD-700-524-1; Roithner Lasertechnik GmbH, Vienna, Austria) and constantly illuminated for different periods of time (5, 15, 60, 120 min). SEAP levels were quantified in the culture supernatant after 48 h.

### Optogenetic remote control of transgene expression in mice

Subcutaneous implants were produced by seeding 5 × 10^4^ pSO3-, pSO4- and pSTING-transgenic HEK-293T cells into 2.5-cm CellMax hollow-fibre membranes (Spectrum Laboratories Inc., Rancho Dominguez, CA, USA) and heat-sealing both ends using a Webster smooth needle holder (Harvard Apparatus, Holliston, MA, USA; cat. no. 512467). Following dorsal subcutaneous implantation into short-term isoflurane-anaesthetized wild-type mice (Oncins France souche 1, Charles River Laboratories, Lyon, France), the animals were directly illuminated for 2 h using a 4 × 8 LED (690 nm, 18 mW sr^−1^; Infors, Bottmingen, Switzerland) placed 10 cm above the standard animal cage. After 48 h, the animals were killed, blood samples were collected and the serum was isolated using microtainer SST tubes according to the manufacturer’s protocol (Becton Dickinson, Plymouth, UK). Serum SEAP levels were then quantified as described above.

### Mind-controlled transgene expression

The synthetic mind-genetic interface that allows mind-controlled transgene expression in a living organism requires different serially linked electronic, optic and genetic components: (i) The BCI (Fig. 5a,b; Supplementary Fig. 5a,b) captures brain waves, processes these electronic signals and provides a, mental state-based (biofeedback, concentration, meditation) electronic output that switches the (ii) field generator ON and OFF (Fig. 5c; Fig. 6e; Supplementary Fig. 5c). The transmitter coil (TC; Fig. 5d; Supplementary Figs 5d, 9 and 10) of the field generator produces an alternating electromagnetic field that inductively couples with the receiver coil (Fig. 5d,e; Fig. 6b; Supplementary Fig. 5d,e) to wirelessly power and programme the (iii) wireless-powered optogenetic implant (Fig. 5e; Fig. 6a–c; Supplementary Fig. 5e) to switch transgene expression of the designer cells inside ON and OFF in a light-dependent and mind-controlled manner.

### BCI

We used a standard commercial low-cost BCI headset (MindSet; NeuroSky Inc., San Jose, USA), which digitizes the brain-wave EEG^53^,^54^,^55^,^56^ (Fig. 5a; Supplementary Fig. 5a). This headset places one dry EEG sensor on the left forehead (two centimeters above the eyebrow) targeting the frontal cortex where cognitive signals linked to higher states of consciousness originate as well as three dry reference electrodes on the left ear and records the following EEG-based information: raw EEG (1 Hz, analogue-to-digital conversion rate), signal quality (0, good signal; 1, poor signal level; off-head state of the EEG sensor); EEG delta band (0.5–2.75 Hz); EEG theta band (3.5–6.75 Hz); EEG low alpha band (7.5–9.25 Hz); EEG high alpha band (10–11.75 Hz); EEG low beta band (13–16.75 Hz); EEG high beta band (18–29.75 Hz); EEG low gamma band (31–39.75 Hz); EEG mid gamma band (41–49.75 Hz). The headset’s microprocessor executes a proprietary algorithm computing a fast Fourier transformation to convert a wide spectrum of brain waves in both time and frequency domains including alpha and beta waves into attention (emphasis on beta waves indicating the user’s mental focus, 14–30 Hz) and meditation (emphasis on alpha waves indicating the user’s mental calmness, 7.5–12 Hz)–meter values (integer values 0–100) that are corrected for eye movement (eye-blinking score, integer value 0–255) and filtered for noise resulting from head movements and muscle artifacts^57^,^56^. The collected data sets are transmitted via Bluetooth (raw data, ms^−1^; processed data, s^−1^) for storage, display on a screen or control of the optogenetic device and wireless-powered implant (see below).

For all mind-control experiments, the subjects sat in a comfortable chair in front of an LCD computer screen wearing the BCI headset and keeping the eyes open at all times. The LCD screen was controlled by a Laptop computer connected to the headset through a bluetooth serial connection. The subjects were verbally instructed to generate three different mental states: biofeedback, concentration and meditation. To generate the biofeedback mental state, the subject was asked to watch the meditation-meter values displayed on a screen and self-train to keep the meditation-meter values above and below a desired threshold. To reach the mental state of concentration, the subject was playing the computer game minesweeper, and for meditation, the subjects were asked to breathe deeply while looking at a landscape still picture on the LCD screen. Unlike for the biofeedback, the subjects did not train to produce mental states of concentration and meditation, as they did not obtain real-time feedback on the screen about their mental states.

The meditation-meter values of the human subjects were transferred from the BCI headset to the Arduino single-board microcontroller (Arduino Uno, Dangi Internet Electronics, Granada, Spain; http://developer.neurosky.com/docs/doku.php?id=mindwave_mobile_and_arduino) in a serial data stream via Bluetooth (BlueSMiRF Gold Bluetooth modem WRL-10268 (SparkFun Electronics, Boulder, CO, USA)) programmed with media access control software and the *CleanProgramBlueSMiRF.pde* script (NeuroSky Modified by Sean Montgomery; www.developer.neurosky.com; Fig. 5b; Supplementary Fig. 5b). The programme running on the Arduino single-board microcontroller (*MindSETArduinoReader.pde*; NeuroSky modified by Sean Montgomery; www.developer.neurosky.com) converted the meditation-meter values into 10 discrete levels, which were visualized using a 10-LED bar along with control LEDs indicating error and signal quality (Fig. 5b; Supplementary Fig. 5b). The data stream could be collected by a computer using the Arduino single-board microcontroller’s serial port running the *MindSETArduinoViewer.pde* processing script (NeuroSky modified by Sean Montgomery; www.developer.neurosky.com) and used to directly switch a NIR LED panel (see above) or the field generator ON or OFF for a specific period of time via a multifunctional USB time-relay device interface (cat. no. 1190035; H-TRONIC, Hirschau, Germany; Fig. 5b; Supplementary Fig. 5b).

To validate the response dynamics of the BCI in cell culture, pSO3/pSO4/pSTING-transgenic HEK-293T cells (5 × 10^4^ cells per well of a 24-well plate) were exposed to mind-controlled illumination by the NIR LED panel, and the resulting SEAP production was profiled in the culture supernatant after 24 h. The human subject wearing the BCI headset performed three different mental states: a self-trained biofeedback mental state (maintaining the observed meditation-meter values on the 10-LED indicator within a desired range); a concentration-based mental state (computer gaming); and a meditation-based mental state (relaxation), all of which were integrated and converted to threshold-dependent activation of the time-delay relay that switched the NIR LED panel ON for a defined period of time (biofeedback: integration, 5 min; threshold, meditation-meter value 90; LED panel activation, 15 min; concentration and meditation: integration, 15 min; threshold, meditation-meter values 80; LED panel activation, 15 s).

### The field generator

The case and the flat coil (FC) (Supplementary Fig. 9) were derived from an induction cooker (IKBE-BT-350KC, Kibernetik AG, Buchs, Switzerland; Fig. 5c; Fig. 6e; Supplementary Figs 5c and 9). The FC contained 21 turns (50 mm inner and 180 mm outer diameter) of a copper thread assembled from 50 parallel 0.35-mm copper wires to minimize electrical resistance (Supplementary Fig. 9). Eight rectangular (50 × 18 × 5 mm) ferrite bars were astrally fixed at the bottom of the FC to guide the field lines and increase the magnetic efficiency (Supplementary Fig. 9). To construct the TC, the FC was connected to a parallel capacitor, a power-managing metal-oxide-semiconductor field-effect transistor (MOSFET) and a pulse-producing synthesized function generator setting the circuit to a frequency of 55 kHz (Supplementary Fig. 10). To maintain the TC in resonance, it was connected to a resonance detection circuit, which feedback controlled the MOSFET. The energy for the resonance detection circuit (15 V d.c.) and MOSFET (63 V d.c.) was provided by a power supply. The TC received its instructions from the BCI via an enable circuit (Supplementary Fig. 5). The TC was fitted into the induction-cooker casing and used as the field generator to produce the magnetic field powering and remote-controlling the wireless-powered optogenetic implant.

### Wireless-powered optogenetic implant

The wireless-powered optogenetic implant was a fully sealed, all-in-one biocompatible device comprising a power receiver, which was remotely powered by electromagnetic induction controlled by the field generator, and the 700-nm NIR LED (*λ*_max_=700 nm, 20 mW sr^−1^; cat. no. ELD-700-524-1; Roithner Lasertechnik, Vienna, Austria), which enabled light-programmable transgene expression of designer cells inside the semi-permeable cultivation chamber (Fig. 5e; Fig. 6a–c; Supplementary Fig. 5e). The power receiver’s antenna was assembled from three orthogonal copper coils (0.1-mm copper wire with 130 windings on a 7 × 7 × 7 mm ferrite cube), three in-series resonance capacitors and six Schottky diodes, which integrated and rectified the current of the three coils and powered the NIR LED in an orientation- and motion-independent manner (Fig. 5d,e; Fig. 6b; Supplementary Figs 5d,e and 11). The entire power receiver, including the base of the NIR LED, was moulded into a spherical polycarbonate cap containing polydimethylsiloxane (PDMS; cat. no. 701912-1, Sigma-Aldrich, Buchs, Switzerland) and fitted to a custom-adapted 500-μl polycarbonate chamber (0.4 × 0.9 mm) with semi-permeable polyethersulfone <300 kDa-cutoff membranes (PES Membrane, VS0651, Sartorius Stedim Biotech, Germany) on two sides (Fig. 5e; Fig. 6a; Supplementary Fig. 5e). The device was sealed by polymerizing the PDMS for 30 min at 50 °C. The coupling intensity of the wireless-powered optogenetic implant was profiled in the space above the field generator by scoring the wireless transmission of power to the implant (Supplementary Fig. 6). A total of 500 μl of a pSO3/pSO4- or pSO3/pSBC-2 (negative control)-transgenic HEK-293F cell suspension (1 × 10^6^ cells) was loaded via a syringe through a hole in the polycarbonate side of the culture chamber, which was sealed with a PDMS plug before implanting the device subcutaneously into the mouse.

### Mind-controlled transgene expression in mice

Cell-containing wireless-powered optogenetic implants were subcutaneously implanted on the backs of short-term isoflurane-anaesthetized wild-type mice (Oncins France souche 1, Charles River Laboratories, Lyon, France), and the cage containing the treated animals was placed on the field generator connected to the BCI. The human subject wearing the BCI headset conducted three different mental states, biofeedback, concentration and meditation, which were integrated (5/25/25 min) and converted to threshold (meditation-meter values 90/75/75)-dependent activation of the time-delay relay that switched the NIR LED in the wireless-powered optogenetic implant ON for defined periods of time (60 min/30 s/30 s) and induced light-triggered SEAP expression in the implanted cells. After 48 and 144 h, blood samples were collected retro-orbitally, and serum SEAP levels were determined as described above. The implants of one treatment group were removed after SEAP profiling at 48 h, and the serum SEAP levels were quantified again 96 h after implant removal. Control mice received wireless-powered optogenetic implants containing pSO3/pSBC-2-transfected HEK-293F cells. Throughout the entire animal study, five 4-week-old female Oncin Souche 1 wild-type mice of the delivered pool were randomly allocated to the individual treatment groups. Neither samples nor animals were excluded from the study and blood-sample analysis was blinded. All experiments involving animals were performed according to the directives of the European Community Council (2010/63/EU), approved by the French Republic (no. 69266310), and performed by Marie Daoud-El Baba at the Institut Universitaire de Technology, IUTA, F-69622 Villeurbanne Cedex, France.

## Author contributions

M.Fo. and M.F. designed the project, analysed the results and wrote the manuscript. M.Fo., S.O., K.Z., T.T., J.H. and M.C. performed the experimental work and analysed the results. M.D.E.-B. performed the mouse work. M.Fo. and P.B. designed, constructed and assembled the electronic components.

## Additional information

**Accession codes**: Sequence information of key components is available at GenBank: human codon-optimized DGCACC3285, GenBank ID: KM591193; human codon-optimized PDEyahA, GenBank ID: KM591194; human codon-optimized PDETBD1265, GenBank ID: KM591195; human codon-optimized DGCL, GenBank ID: KM591196; PhIFN_hIFNβ_, GenBank ID: KM591197; PIFN(AC+), GenBank ID: KM591198; PIFN(AC+).

**How to cite this article**: Folcher, M. *et al.* Mind-controlled transgene expression by a wireless-powered optogenetic designer cell implant. *Nat. Commun.* 5:5392 doi: 10.1038/ncomms6392 (2014).

## Supplementary Material

Supplementary Information Supplementary Figures 1-11, Supplementary Table 1 and Supplementary References
