# Supplementary material for: Mind-controlled transgene expression by a wireless-powered optogenetic designer cell implant
Source: Nat Commun. 2014 Nov 11;5:5392. doi: 10.1038/ncomms6392 (PMC4241983; doi:10.1038/ncomms6392)
Supplement: Supplementary Information — Supplementary Figures 1-11, Supplementary Table 1 and Supplementary References [file ncomms6392-s1.pdf]

Mind-controlled transgene expression by a wireless-powered optogenetic designer cell implant

Marc Folcher[1], Sabine Oesterle[1], Katharina Zwicky[1], Thushara Thekkottil[1], Julie Heymoz[1], Muriel Hohmann[1], Matthias Christen[1], Marie Daoud El-Baba[2], Peter Buchmann[1], Martin Fussenegger[1,3]

Correspondence should be addressed to M.F. (e-mail: fussenegger@bsse.ethz.ch)

Supplementary Information

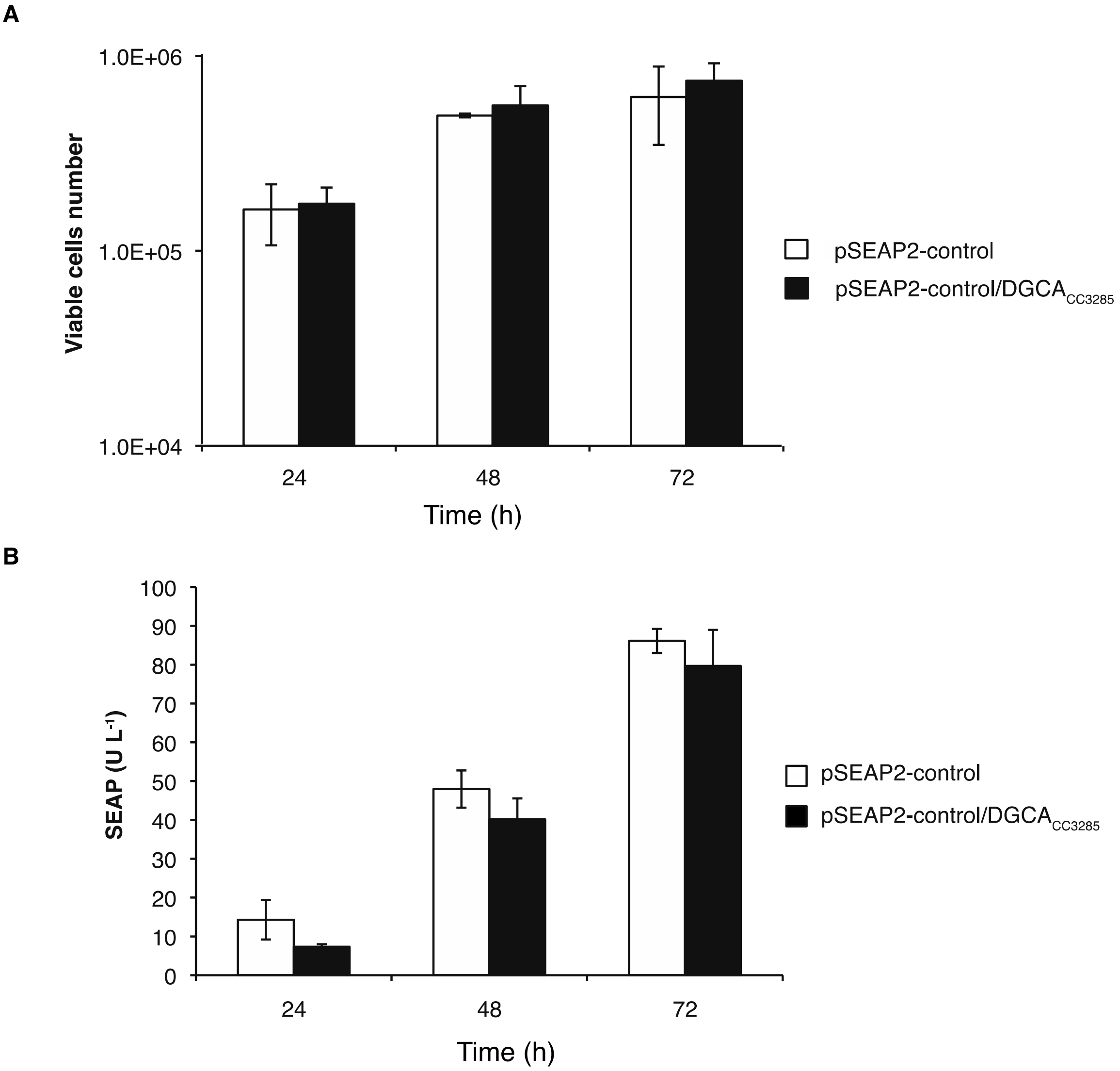

Supplementary Figure 1 | Impact of constitutive diguanylate cyclase expression on the viability and metabolic capacity of mammalian cells. 5x10^5 HEK-293T were cotransfected with the constitutive *Caulobacter crescentus* diguanylate cyclase A (DGCA$_{CC3285}$) expression vector pZKY121 (P$_{SV40}$-DGCA$_{CC3285}$-pA) and the constitutive SEAP expression vector pSEAP2-control (P$_{SV40}$-SEAP-pA) and cell viability (**a**) as well as the SEAP production capacity (**b**) were profiled for up to 72h. Data are mean ± SD; n=5, triplicate experiments.

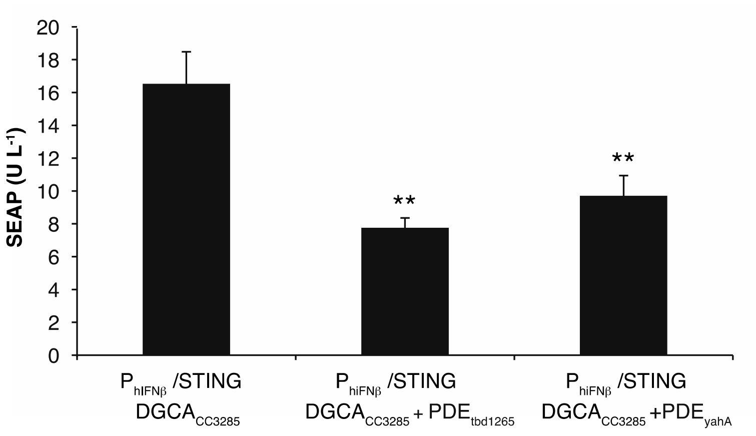

Supplementary Figure 2 | Impact of constitutive PDE expression on the synthetic c-di-GMP-specific second messenger pathway. $5x10^5$ HEK-293T containing pZKY121, pSTING and pSO1 were individually (co-)transfected with the c-di-GMP-specific phosphodiesterases PDE_{yahA} (pZKY119; P_{SV40}-PDE_{yahA}-pA) or $PDE_{TBD1265}$ (pZKY120; P_{SV40}-$PDE_{TBD1265}$-pA) and SEAP expression was scored in the culture supernatant after 48h. Constitutive expression of either phosphodiesterase (PDE) reduced c-di-GMP-specific STING-mediated activation of $P_{hIFNß}$-driven SEAP expression. Data are mean ± SD; statistics by two-tailed t test in comparison to control ($P_{hIFNβ}$/ STING, $DGCA_{CC3285}$); n=5, triplicate experiments, $**P < 0.01$.

$P_{hIFN\beta}$	(pSO1)	ATGACAGAGGAAAACTGAAAGGGAGAACTGA
$P_{IFN(AC+)}$	(pSO2)	ATGACAAAGGGAAACTGAAAGGGAAAACTGA
$P_{IFN(ACD+)}$	(pSO3)	ATGACAAAGGGAAACTGAAAGGGAAA-CTGA

Supplementary Figure 3 | Interferon-β promoter variants. Sequence alignment showing IRF3-optimised operator sites in different human interferon-β ($P_{hIFN\beta}$) promoter variants. Point mutations and deletions are shown in red.

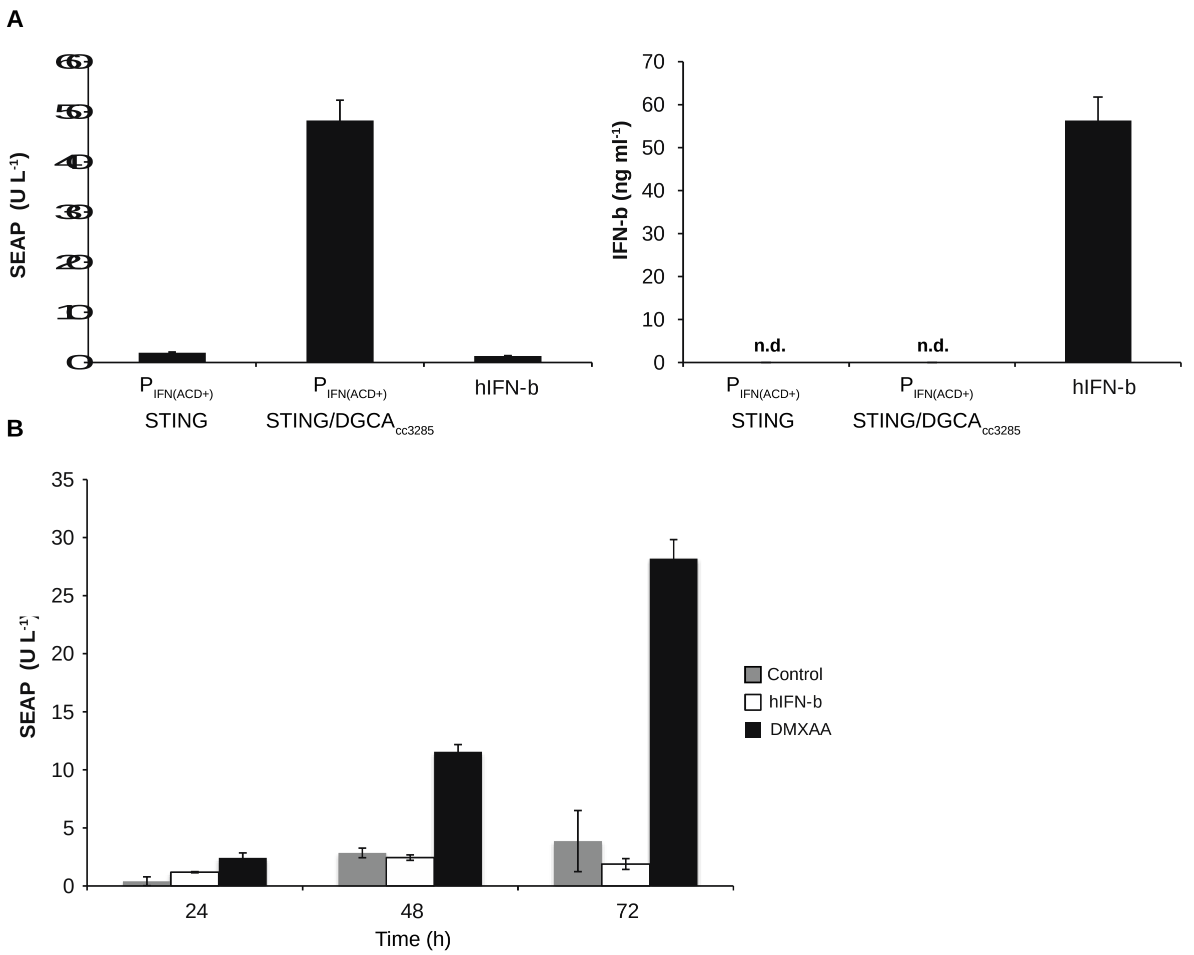

Supplementary Figure 4 | Analysis of potential interference of the orthogonal c-di-GMP-based second messenger signalling pathway with hIFN-β production and paracrine hIFN-β signalling. (**a**) The orthogonal c-di-GMP-based second messenger signalling pathway fails to induce endogenous hIFN-β secretion in engineered HEK-293T. 5x10^4 HEK-293T cells were cotransfected with pSTING (P_{hCMV}-STING-pA) and pSO3 ($P_{IFN(ACD+)}$-SEAP-pA) as well as optionally with pZKY121 (P_{SV40}-$DGCA_{CC3285}$-pA) and SEAP as well as hIFN-β levels were profiled in the culture supernatant after 48h. HEK-293T transfected with the constitutive hIFN-β expression vector pWW512 ($P_{hEF1\alpha}$-hIFN-β-pA) were used as positive control. (**b**) Paracrine hIFN-β has no impact on the orthogonal c-di-GMP-based second messenger signalling pathway. 5x10^4 HEK-293T cells were cotransfected with pSTING (P_{hCMV}-STING-pA) as well as pSO3 ($P_{IFN(ACD+)}$-SEAP-pA) and exposed to recombinant hIFN-β for 48h before SEAP expression was profiled in the culture supernatant. The STING-dependent type I interferon inducer DMXXA was used as positive control. Data are mean ± SD; n=5, triplicate experiments.

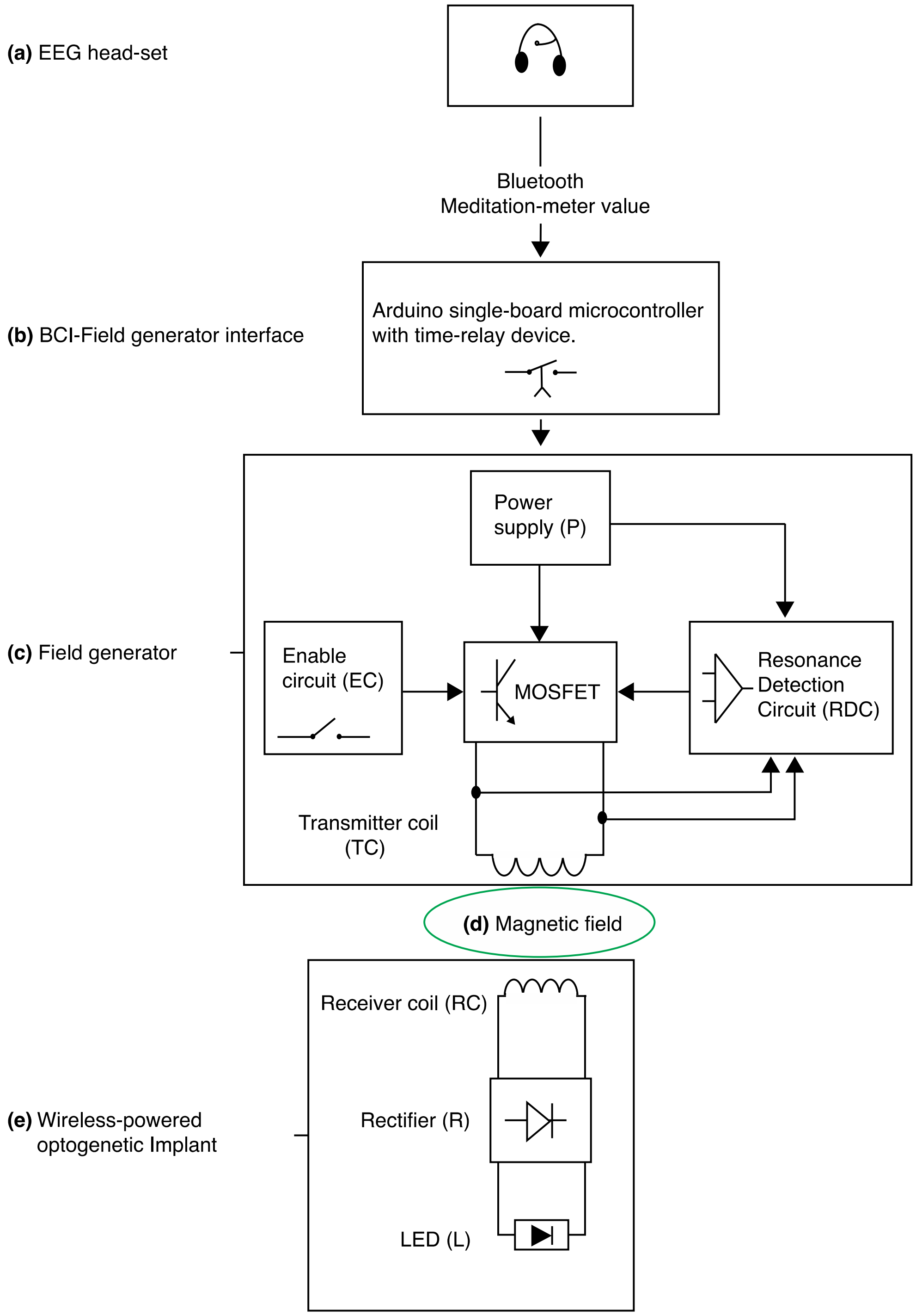

Supplementary Figure 5 | Schematic of the electronic components of the mind-controlled transgene expression device. Electronic wire diagram of the electronic components shown in Figure 5 of the main text: (**a**) Electroencephalography (EEG) head-set, (**b**) Arduino single-board microcontroller, (**c**) transmitter-coil (TC)-containing field generator (FG), (**d**) inductively linked receiver coil-containing (**e**) wireless powered optogenetic implant.

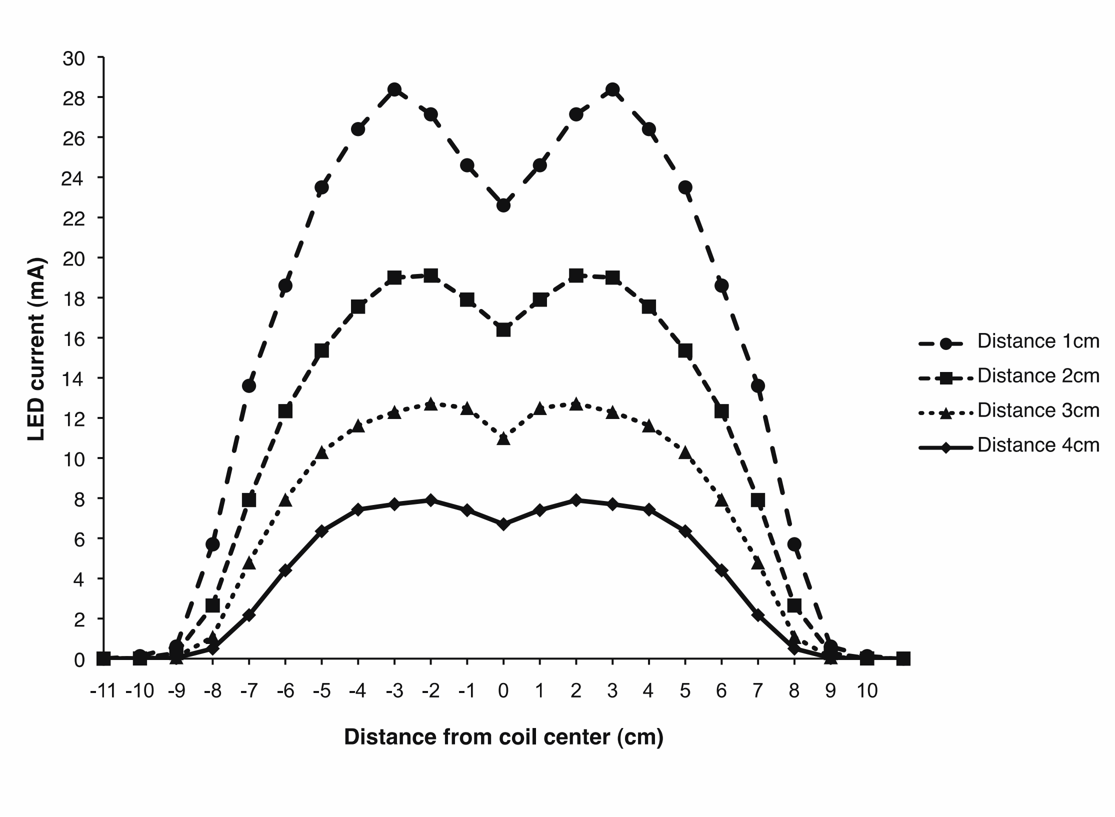

Supplementary Figure 6 | Distance-dependent coupling intensity of the wireless-powered optogenetic implant and the field generator. Measurement of the NIR-LED of the wireless-powered optogenetic implant in the space above the field generator.

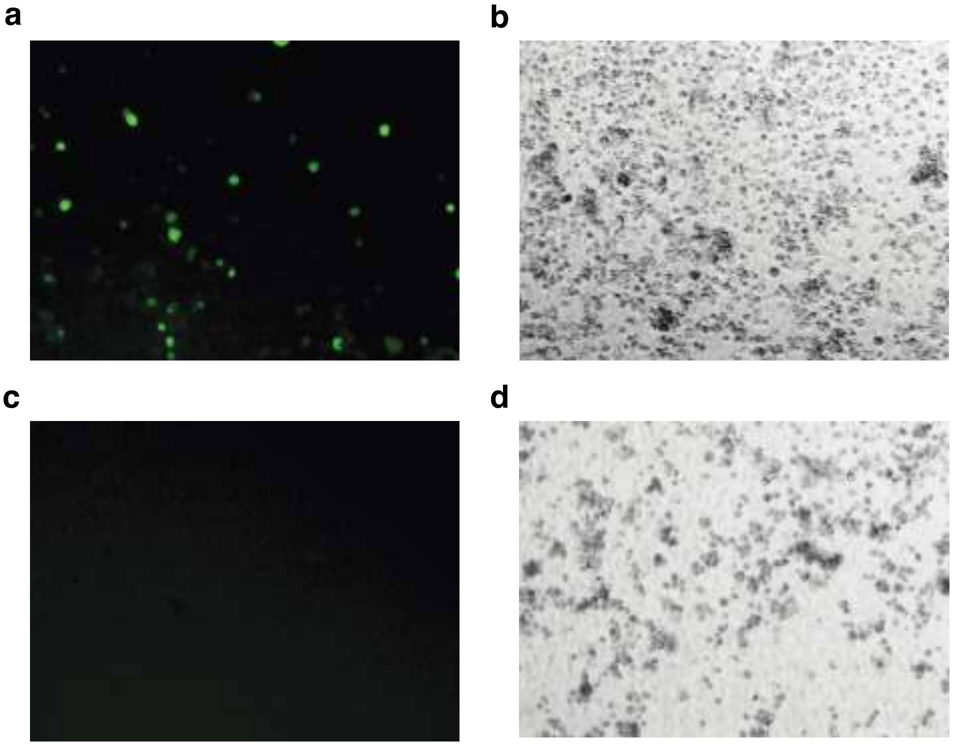

Supplementary Figure 7 | Virus permeability of the wireless-powered optogenetic implant's cultivation chamber. Fluorescence (**a**, **c**) band bright-field (**b**, **d**) micrographs of HEK-293F cell grown inside (**a**) and outside (**c**) of the wireless-powered optogenetic implant 72h after infection of the inside population with EYFP-transducing lentiviral particles. Due to the <300kDa molecular cut-off lentiviral particles are unable to cross the semi-permeable polyethersulfone membrane of culture chamber.

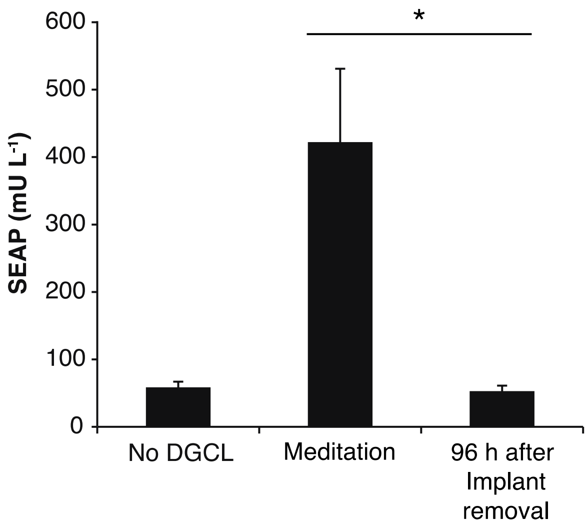

Supplementary Figure 8 | Blood SEAP levels before and after surgical removal of the wireless-powered optogenetic implants. The wireless-powered optogenetic implants of mice whose blood SEAP levels were controlled by a human subject's meditation (relaxation) were surgically explanted after 48h the serum SEAP levels were again profiled 4 days later. The identical treatment group carrying the implant for the entire period of 6 days and isogenic animals containing DGCL-deficient implants were used as controls. Data are mean ± SD; statistics by two-tailed t test; n=5 mice. *$P < 0.05$.

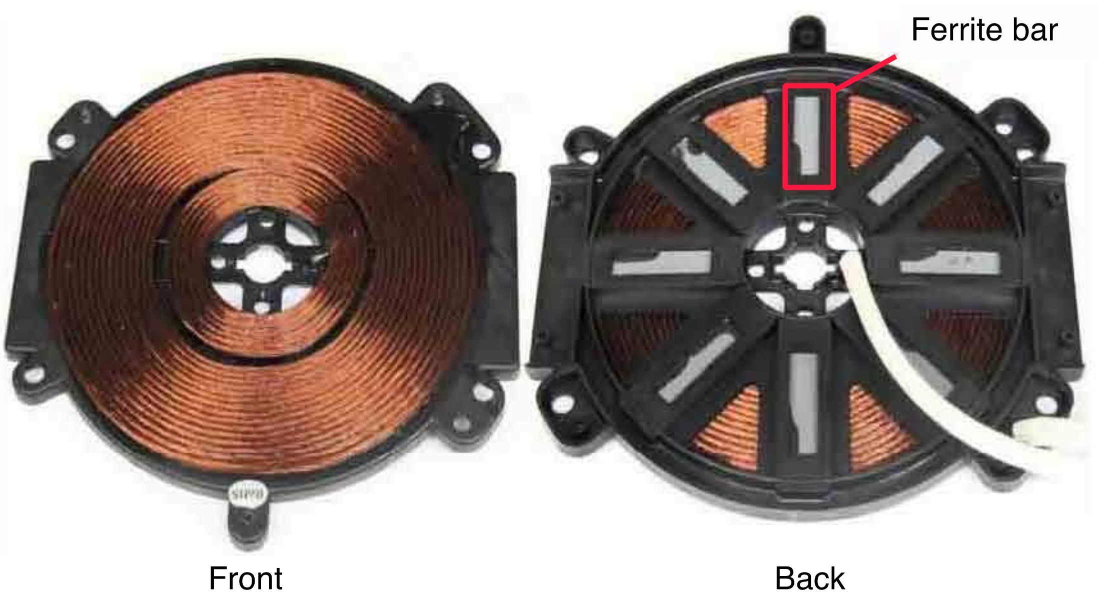

Supplementary Figure 9 | Flat coil. Front and back view of the flat coil containing 21 turns of a copper thread assembled from 50 parallel 0.35mm copper wires to minimize electrical resistance and 8 astrally fixed rectangular ferrite bars at the bottom of the flat coil to guide the field lines and increase magnetic efficiency.

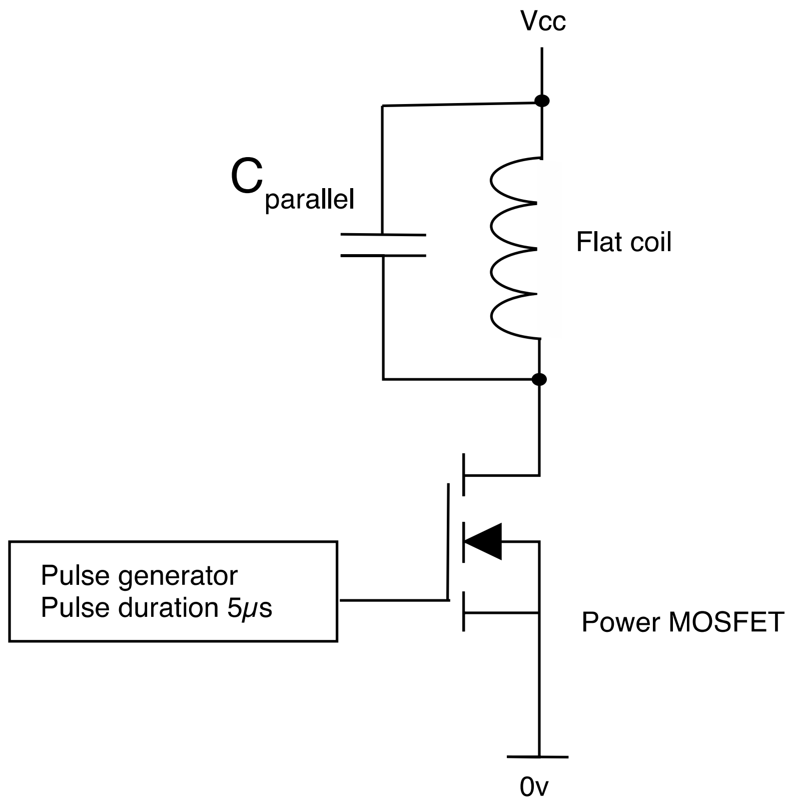

Supplementary Figure 10 | Schematic of the transmitter-coil circuit. The transmitter coil (TC) consists of a flat coil connected to a parallel capacitor, a power-managing metal-oxide-semiconductor field-effect transistor (MOSFET) and a pulse-producing synthesized function generator (FG).

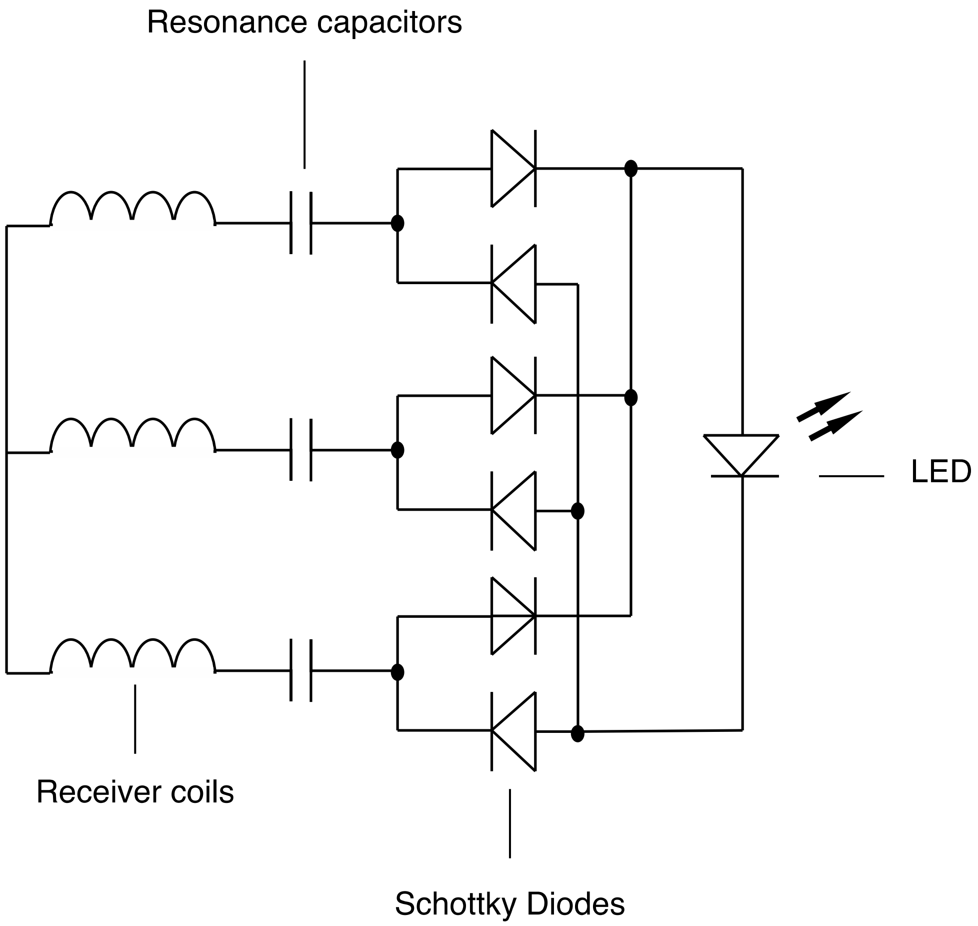

Supplementary Figure 11 | Schematic of the receiver circuit. Three orthogonal receiver coils connected to three in-series resonance capacitors and a rectifier circuit composed of six Schottky diodes powering the NIR-light LED**.**

Supplementary Table 1. Plasmids and oligonucleotides used and designed in this study

Plasmid	Description and Cloning Strategy	Reference or Source
pcDNA3.1(+)	Constitutive P_{hCMV}-driven mammalian expression vector (P_{hCMV}-MCS-pA).	Invitrogen
pCD/NL/BH*	HIV-1-derived GAG/Pol/TAT-encoding helper plasmid (P_{hCMV}-GAG-Pol-TAT-pA).	(*1*)
pET15b::mYPet-ycgR-mCyPet	Consitutive prokaryotic expression vector encoding mYPet-YcgR-mCyPet (P_{T7}-mYPet-YcgR-mCyPet).	(*2*)
pGEM®-T Easy	Vector for cloning of PCR products.	Promega
pLTR-G	Constitutive mammalian VSV-G expression vector (5'LTR-VSV-G-pA).	(*3*)
pMM1	Constitutive pcDNA3.1(+)-derived expression vector with modified MCS (P_{hCMV}-MCS-pA; MCS, *Eco*RI-ATG-*Spe*I-*Nhe*I-*Bam*HI-STOP-*Xba*I-*Hind*III-*Fse*I-pA). pcDNA3.1 was PCR-amplified using oligonucleotides OMM1 (5-cggatccaccggtgtctagaaagctttgaggccggcctgcaggGATCAGCCTCGACTGTGCCTTC-3') and OMM2 (5'-ctagcactagtcatggtgaattcgattaatcaattgacgcgtGGAGATCTCCCGATCCGTC-3') and self-ligated.	Mueller et al., unpublished
pNLK8	HIV-1-derived constitutive lentiviral EYFP expression vector (5'LTR-ψ^+-ori_{SV40}-cPPT-RRE-$P_{hEF1\alpha}$-EYFP-3'$LTR_{\Delta U3}$).	(*4*)
pSBC-2	Constitutive mammalian expression vector (P_{SV40}-MCS-pA).	(*5*)
pSEAP2-control	Constitutive mammalian SEAP expression vector (P_{SV40}-SEAP-pA).	Clontech
pWW512	Constitutive mammalian hIFN-β expression vector ($P_{hEF1\alpha}$-hIFN-β-pA).	(*6*)
pSTING	Constitutive mammalian STING expression vector (P_{hCMV}-STING-pA).	IMAGE: IRAVp968F0688D
pUC57	pUC19-derived prokaryotic expression vector.	Genescript
pMFO1	Custom-designed pUC57-derived vector containing the human codon-optimized $PDE_{TBD1265}$.	This work
pMFO2	Custom-designed pUC57-derived vector containing the human codon-optimized $DGCA_{CC3285}$.	This work
pMFO4	Custom-designed pUC57-derived vector containing the human codon-optimized PDE_{yahA}.	This work
pMFO5	Custom-designed pUC57-derived vector containing the human codon-optimized DGCL.	This work
pMFO6	Custom-designed pUC57-derived vector containing the human codon-optimized mYPet-YcgR-mCyPet.	This work

pKZY81	Constitutive mammalian mYPet-YcgR-mCyPet expression vector (P_{SV40}-mYPet-YcgR-mCyPet-pA). mYPet-YcgR-mCyPet was excised from pMFO6 using *Eco*RI/*Pst*I and cloned into the corresponding sites (*Eco*RI/*Pst*I) of pSBC-2.	This work
pKZY113	Constitutive mammalian DGCL expression vector (P_{SV40}-DGCL-pA). DGCL was excised from pMFO5 using *Eco*RI/*Pst*I and cloned into the corresponding sites (*Eco*RI/*Pst*I) of pSBC-2.	This work
pKZY119	Constitutive mammalian PDE_{yahA} expression vector (P_{SV40}-PDE_{yahA}-pA). PDE_{yahA} was excised from pMSO2 using *Eco*RI/*Pst*I and cloned into the corresponding sites (*Eco*RI/*Pst*I) of pSBC-2.	This work
pKZY120	Constitutive mammalian $PDE_{TBD1265}$ expression vector (P_{SV40}-$PDE_{TBD1265}$-pA). $PDE_{TBD1265}$ was excised from pMFO1 using *Eco*RI/*Pst*I and cloned into the corresponding sites (*Eco*RI/*Pst*I) of pSBC-2.	This work
pKZY121	Constitutive mammalian $DGCA_{CC3285}$ expression vector (P_{SV40}-$DGCA_{CC3285}$-pA). $DGCA_{CC3285}$ was excised from pMFO2 using *Eco*RI/*Pst*I and cloned into the corresponding sites (*Eco*RI/*Pst*I) of pSBC-2.	This work
pSO1	$P_{hIFN\beta}$-driven SEAP expression vector ($P_{hIFN\beta}$-SEAP-pA). $P_{hIFN\beta}$ was PCR-amplified from HEK-293 genomic DNA using oligonucleotides OSO1 (5'-aatgagctagcgaattcTCAGGTCGTTTGCTTTC-3') and OSO2 (5'atgaaagcttcATGTTGACAACACGAACAGTG-3') restricted with *Nhe*I/*Hin*dIII and cloned into the corresponding site (*Nhe*I/*Hin*dIII) of pSEAP2-control.	This work
pSO2	$P_{IFN(AC+)}$-driven SEAP expression vector ($P_{IFN(AC+)}$-SEAP-pA). The IRF3 operator sites of $P_{hIFN\beta}$ were sequence optimized by PCR-based site-directed mutagenesis using the oligonucleotides OSO3 (5'-AaAGGgAAACTGAAAGGGAaAACTGAAAGTGGGAAATTCCTCTG-3') and OSO4 (5'-GTCATT TACATTTTAGTAGTTTC-3') and pSO1 as template.	This work
pSO3	$P_{IFN(ACD+)}$-driven SEAP expression vector ($P_{IFN(ACD+)}$-SEAP-pA). The IRF3 operator sites of $P_{IFN(AC+)}$ were space optimized by PCR-based site-directed mutagenesis using the oligonucleotides OSO5 (5'-AAAGGG AAACTGAAAGGGAAACTGAAAGTGGGAAATTCCTCTG -3') and OSO4 (5'-GTCATTTACATTTT AGTAGTTTC-3') and pSO2 as template.	This work
pSO4	Constitutive mammalian DGCL expression vector (P_{hCMV}-DGCL-pA). DGCL was excised from pKZY113 using *Eco*RI/*Hin*dIII and cloned into the corresponding sites (*Eco*RI/*Hin*dIII) of pMM1.	This work

Oligonucleotides: restriction endonuclease-specific sites are underlined in oligonucleotide sequences. Annealing base pairs contained in oligonucleotide sequences are shown in capital letters.

Abbreviations: **3'LTR$_{\Delta U3}$**, HIV-1-derived enhancer-free 3'LTR variant; **5'LTR**, HIV-1-derived 5' long terminal repeat; **c-di-GMP**, cyclic diguanosine monophosphate; **cPPT**, central polypurine tract; **DGCA$_{CC3285}$**, feedback-inhibited CC3285 diguanylate cyclase A of *Caulobacter crescentus* (ATCC 19089/CB15) (human codon-optimized $DGCA_{CC3285}$, GenBank ID: KM591193) (*7*); **DGCL**, Near infrared-light-activated diguanylate cyclase derived from *Rhodobacter sphaeroides* BphG1 (ATCC BAA-808; N-terminal PAS-GAF-PHY-GGDEF portion of BphG1 (Q8VRN4_RHOSH) and the catalytic diguanylate cyclase domain GGDEF which is photo-activated by it's cognate PAS-GAF-PHY phytochrome) (human codon-optimzed DGCL, GenBank ID: KM591196 (*8*); **EGFP**, enhanced GFP; **EYFP**, yellow-fluorescent GFP variant; **GFP**, enhanced green-fluorescent protein; **GAG**, HIV-derived core protein; **hIFN-β**, human interferon beta; **HIV**, human immunodeficiency virus; **IRF3**, interferon regulatory factor 3; **pA**, polyadenylation signal; **MCS**, multiple cloning site; **mYPet-**

YcgR-mCyPet, N-terminally His6-tagged c-di-GMP biosensor consisting of a central *Salmonella typhimurium*-derived diguanylate receptor domain (YcgR) flanked by yellow (mYPet) and cyan (mCYPet) fluorescent protein domains; **ori_{SV40}**, simian virus 40-derived origin of replication; **PDE_{yahA}**, cyclic diguanylate (c-di-GMP)-degrading phosphodiesterase of *Escherichia coli* (ATCC 8937) (human codon-optimized PDE_{yahA}, GenBank ID: KM591194 (*9*); **$PDE_{TBD1265}$**, cyclic diguanylate (c-di-GMP)-degrading phosphodiesterase of *Thiobacillus denitrificans* (ATCC 25259) (human codon-optimized $PDE_{TBD1265}$, GenBank ID: KM591195)(*10*); **P_{hCMV}**, human cytomegalovirus immediate early promoter; **$P_{hCMVmin}$**, minimal version of P_{hCMV}; **$P_{hEF1\alpha}$**, human elongation factor 1 alpha promoter; **$P_{hIFN\beta}$**, human interferon beta promoter (GenBank ID: KM591197); **$P_{IFN(AC+)}$**, modified $P_{hIFN\beta}$ containing sequence-optimized IRF3 operator sites (GenBank ID: KM591198) (*11*); **$P_{IFN(ACD+)}$**, $P_{IFN(AC+)}$ containing space-optimized IRF3 operator sites (GenBank ID: KM591199) (*25*); **Pol**, HIV virion-associated polymerase; **P_{SV40}**, simian virus 40 promoter; **P_{T7}**, bacteriophage T7 promoter; **RRE**, HIV-1-derived Rev response element; **SEAP**, human placental secreted alkaline phosphatase; **STING**, mouse stimulator of interferon genes; **TAT**, HIV-1-derived transactivator of transcription; **VSV-G**, vesicular stomatitis virus protein G; **ψ^+**, extended HIV-1-derived lentiviral packaging signal.

Suplementary References

1. Mochizuki, H., Schwartz, J.P., Tanaka, K., Brady, R.O. & Reiser, J. High-titer human immunodeficiency virus type 1-based vector systems for gene delivery into nondividing cells. *J. Virol.* **72**, 8873-8883 (1998).
2. Christen, M., Kulasekara, H.D., Christen, B., Kulasekara, B.R., Hoffman, L.R. & Miller, S.I. Asymmetrical distribution of the second messenger c-di-GMP upon bacterial cell division. *Science* **328**, 1295-1297 (2010).
3. Reiser, J., Harmison, G., Kluepfel-Stahl, S., Brady, R.O., Karlsson, S., & Schubert, M. Transduction of nondividing cells using pseudotyped defective high-titer HIV type 1 particles. *Proc. Natl. Acad. Sci. USA* **93**, 15266-15271 (1996).
4. Link, N. *et al.* Therapeutic protein transduction of mammalian cells and mice by nucleic acid-free lentiviral nanoparticles. *Nucleic Acids Res.* **34**, e16 (2006).
5. Nuthmann, V., Dirks, W. & Drexler, H.G. Transient expression of the tartrate-resistant acid phosphatase (TRAP) gene in hamster cells: a pilot study. *Leukemia* **7**, 1960-1964 (1993).
6. Weber, W. *et al.* Gas-inducible transgene expression in mammalian cells and mice. *Nat. Biotechnol.* **22**, 1440-1444 (2004).
7. Christen, B. *et al.* Allosteric control of cyclic di-GMP signaling. *J. Biol. Chem.* **281**, 32015-32024 (2006).
8. Tarutina, M., Ryjenkov, D.A. & Gomelsky, M. An unorthodox bacteriophytochrome from Rhodobacter sphaeroides involved in turnover of the second messenger c-di-GMP. *J. Biol. Chem.* **281**, 34751-34758 (2006).
9. Schmidt, A.J., Ryjenkov, D.A. & Gomelsky, M. The ubiquitous protein domain EAL is a cyclic diguanylate-specific phosphodiesterase: enzymatically active and inactive EAL domains. *J. Bacteriol.* **187**, 4774-4781 (2005).
10. Tchigvintsev, A. *et al.* Structural insight into the mechanism of c-di-GMP hydrolysis by EAL domain phosphodiesterases. *J. Mol. Biol.* **402**, 524-538 (2010).
11. Escalante, C.R., Nistal-Villan, E., Shen, L., Garcia-Sastre. A. & Aggarwal, A.K. Structure of IRF-3 bound to the PRDIII-I regulatory element of the human interferon-beta enhancer. *Mol. Cell* **26**, 703-716 (2007).
